# Soret-Effect Induced Phase-Change in a Chromium Nitride Semiconductor Film

**DOI:** 10.1021/acsnano.4c03574

**Published:** 2024-08-01

**Authors:** Yi Shuang, Shunsuke Mori, Takuya Yamamoto, Shogo Hatayama, Yuta Saito, Paul J. Fons, Yun-Heub Song, Jin-Pyo Hong, Daisuke Ando, Yuji Sutou

**Affiliations:** † WPI Advanced Institute for Materials Research, Tohoku University, 2-1-1 Katahira, Aoba, Sendai 980-8577, Japan; ‡ Department of Materials Science, Graduate School of Engineering, Tohoku University, 6-6-11 Aoba-yama, Sendai 980-8579, Japan; § Department of Metallurgy, Graduate School of Engineering, Tohoku University, Miyagi 980-8579, Japan; ∥ Device Technology Research Institute, National Institute of Advanced Industrial Science and Technology (AIST), Tsukuba Central 2, Umezono 1-1-1, Tsukuba 305-8568, Japan; ⊥ Department of Electronics and Electrical Engineering, Faculty of Science and Technology, Keio University, 3-14-1 Hiyoshi, Kohoku-ku, Yokohama, Kanagawa 223-8522, Japan; # Department of Electronic Engineering, Hanyang University, 17 Haengdang-dong, Seongdong-gu, Seoul 133-791, Korea; ∇ Department of Physics, Hanyang University, Seoul 04763, Korea

**Keywords:** nitride, melting-free, soret-effect, phase-change materials, nonvolatile memory

## Abstract

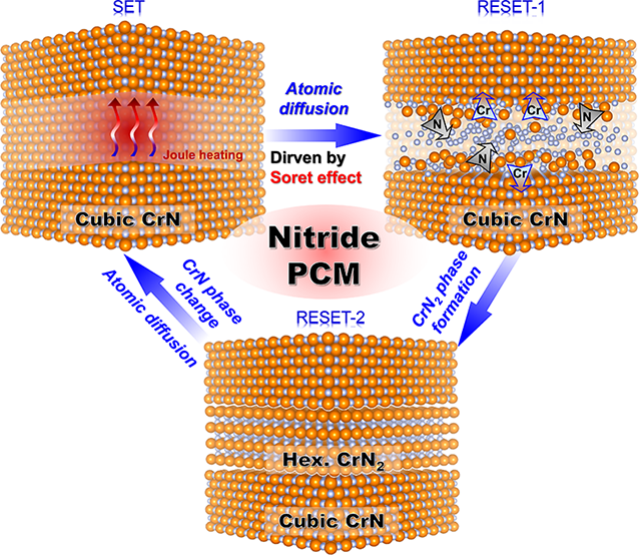

Phase-change materials such as Ge–Sb–Te (GST) exhibiting amorphous and crystalline phases can be used for phase-change random-access memory (PCRAM). GST-based PCRAM has been applied as a storage-class memory; however, its relatively low ON/OFF ratio and the large Joule heating energy required for the RESET process (amorphization) significantly limit the storage density. This study proposes a phase-change nitride, CrN, with a much wider programming window (ON/OFF ratio more than 10^5^) and lower RESET energy (one order of magnitude reduction from GST). High-resolution transmission electron microscopy revealed a phase-change from the low-resistance cubic CrN phase into the highly resistive hexagonal CrN_2_ phase induced by the Soret-effect. The proposed phase-change nitride could greatly expand the scope of conventional phase-change chalcogenides and offer a strategy for the next-generation of PCRAM, enabling a large ON/OFF ratio (∼10^5^), low switching energy (∼100 pJ), and fast operation (∼30 ns).

## Introduction

Phase-change materials (PCMs) represented by Ge–Sb–Te (GST) are mainly chalcogenides and have been widely studied for nonvolatile resistive switching memory due to their fast and reversible phase change between the amorphous and crystalline phases. However, GST faces a fundamental challenge in requiring large Joule heating energy to achieve the melting required for the RESET process (amorphization), which inevitably results in higher operating current and power dissipation.^1^ Furthermore, crystallization from the amorphous phase limits the switching speed of the device. Manipulation of the incubation process has proven to be an effective method of reducing the crystallization time of a PCM through the application of external stimuli, such as an electrical field, or the implementation of alloying strategies, such as doping or stoichiometry control.^2−6^ Advanced concepts such as melt-free crystalline-to-crystalline PCMs are attracting much attention due to their low write powers and fast switching speeds. They include artificial GeTe/Sb_2_Te_3_ superlattices, the so-called iPCM (interfacial phase-change memory) devices, where the resistance contrast between two crystalline phases comes from different bonding states, namely, a covalently bonded state of high resistance and a resonantly bonded state of low resistance.^7^ Another category is polymorphic-change memory devices based on polymorphic compounds such as MnTe, where a phase change occurs via atomic-displacement between NiAs-type and Wurzite-type hexagonal phases, which induces Te coordination changes, leading to a large resistance contrast.^8^ Furthermore, two-dimensional (2D) materials such as In_2_Se_3_ and MoTe_2_ can undergo structural phase-change driven by an external electrical field, exhibiting electrical properties ranging from semiconducting to metallic behavior.^9,10^ Nonetheless, fabricating a superlattice or single-/few-layered 2D film devices is complicated and requires great effort. Moreover, Se- or Te-based materials are not environmentally friendly, and the Te segregation observed after many switching cycles is thought to affect device longevity. In this regard, a nitride memory material can offer better processing and chemical compatibility with nitride electrodes, which are generally used in complementary metal–oxide–semiconductor (CMOS) circuits and can be easily applied to power electronics based on nitrides such as GaN.^11^ In recent years, nitride materials have garnered significant attention in the realm of CMOS-compatible nonvolatile memory (NVM) devices, including ferroelectric diodes (FeD) switching-based memory and resistive random-access memory (ReRAM). Notably, Jariwala et al. achieved success in developing a ferroelectric wurtzite nitride, specifically AlScN, capable of enabling innovative compute-in-memory (CIM) architectures.^12−15^ This material demonstrates a large on/off ratio in addition to good data retention properties. Besides, the switching mechanism of nitrides has predominantly relied on the formation and motion of N vacancies that lead to the creation of conducting filaments in a highly insulating nitride matrix, which is known as ReRAM.^11,16,17^ However, a variety of challenges still remain in the insulating nitride-based memory such as Si_3_N_4_ and AlN,^11,16^ hindering its practical applications. First, a high-energy forming process is necessary to generate conductive filaments arising from vacancies in a virgin device under electric bias, which greatly increases energy consumption.^18^ Second, the number of vacancies or defects in insulators is not easy to be controlled; thus, the switching and failure mechanisms remain unclear.^19^

Unlike the above-mentioned insulating nitrides, CrN is a well-known electronic material with a conducting behavior that can be varied from metallic to semiconductor-like by tuning defects, and it has been widely studied in fields such as supercapacitors and thermoelectrics.^20,21^ Besides, the CrN crystalline phase or structure is easily tuned by external stresses such as pressure,^22^ electron beam irradiation,^23^ or thermal treatment.^24^ Therefore, CrN has a strong potential for applications in phase-change NVM due to its large variation in electrical properties upon structural changes. Here, we propose a CrN memory material that undergoes a nonvolatile and reversible crystalline-to-crystalline phase-change upon application of electrical pulses, enabling environmentally friendly NVM devices with a large programming window (∼10^5^), low power consumption (∼100 pJ), and fast operation speed (∼30 ns).

## Results and Discussions

### Resistive Switching Behavior of CrN Device

**Figure 1:**
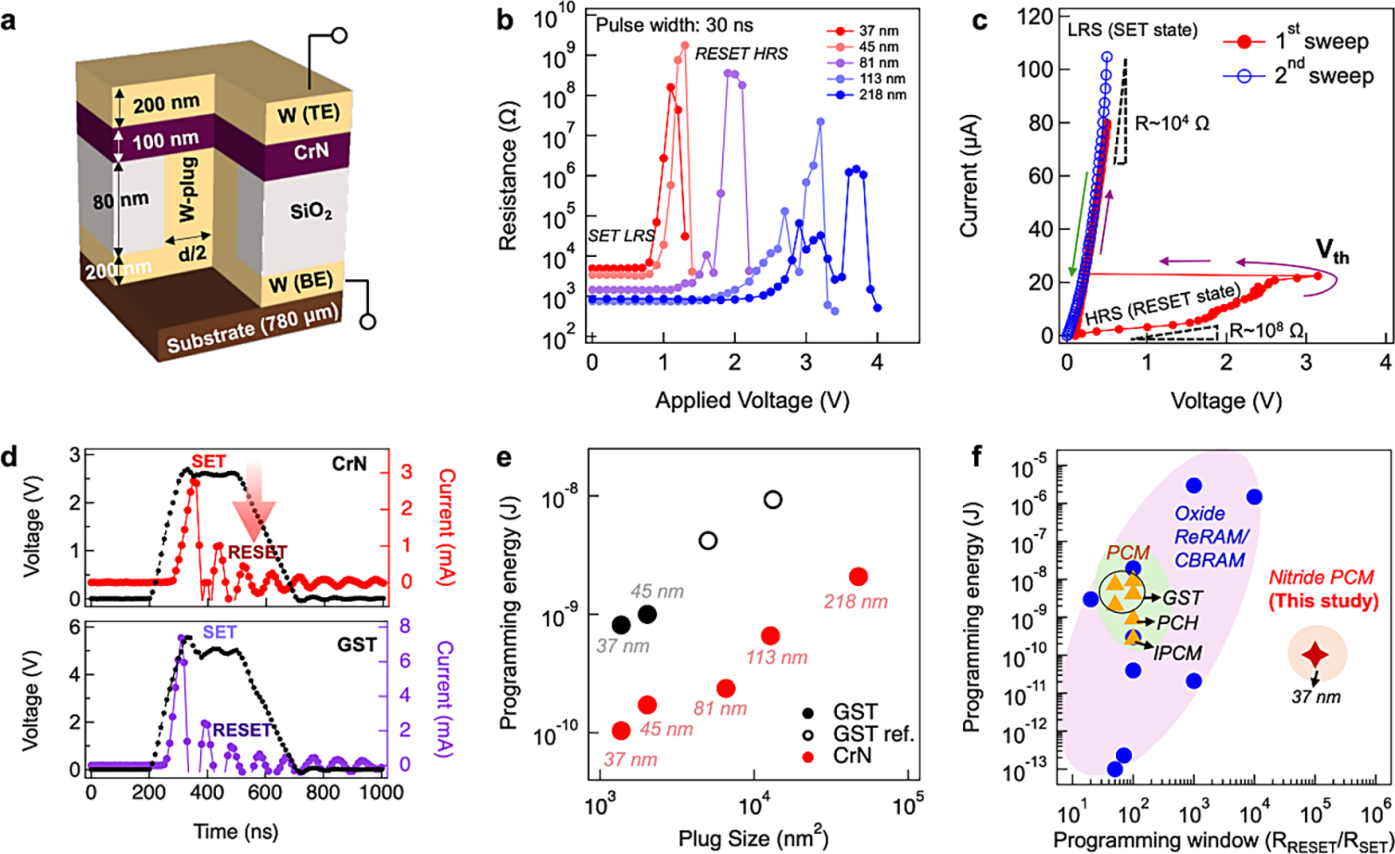
Structure and switching performance of a CrN-based memory device. (a) Cross-section of a conventional T-shaped memory device (*d*: side length of the W plug; TE: top electrode; and BE: bottom electrode). (b) Resistance as a function of the pulse voltage at various plug sizes; the pulse width was fixed at 30 ns, and the read voltage was 0.1 V. (c) Threshold switching behavior, showing a resistance change from ∼10^8^ (high-resistance state: HRS) to ∼10^4^ (low-resistance state: LRS). (d) RESET voltage pulse and its corresponding transient current in CrN and GST devices, with a plug size of 45 × 45 nm^2^, measured using a waveform of 200 ns with a leading-edge time of 100 ns and a trailing-edge time of 200 ns. (e) Contact area dependence of the operation energy for CrN and Ge_2_Sb_2_Te_5_ (GST)-based memory devices; the open markers indicate the GST reference plot for a T-shape device.^4,28,31^ (f) Benchmark plot of programming energy vs. programming window of CrN-PCM with other representative NVM publications.^7,28,31,32^

We fabricated a conventional T-shaped memory (Figure S1), whose cross section is schematized in Figure 1a. It consisted of a square W plug (heater electrode) with a side length *d* varying from 37 to 218 nm. After CrN deposition, the top electrode (TE) was sequentially deposited *in situ* in the same sputtering chamber to avoid surface oxidation of CrN. The CrN layer was characterized by X-ray diffraction (XRD) and transmission electron microscopy (TEM), revealing homogeneous growth with an NaCl-like cubic phase (Figure S2). Based on Rutherford backscattering spectrometry (RBS) measurements, the CrN film was found to include a small amount of unintentionally doped oxygen from the sputtering process and with a measured composition of Cr: 47.5 at %, N: 43.9 at %, and O: 8.6 at %, where the cation/anion composition ratio was 0.91. The as-deposited CrN thin film shows semiconducting behavior as indicated by the temperature dependence of resistivity from Hall measurements (Figure S3). The initial state of the device was the as-deposited low-resistance (∼10^3–4^ Ω) cubic CrN state. The resistance (*R*) was then read while increasing the amplitude of the applied pulse *V* (30 ns width) by 0.1 V (Figure S4). Figure 1b displays the resulting *RV* characteristics. For a device with a plug size of 37 × 37 nm^2^, when a positive voltage pulse was applied to the bottom electrode (BE), the device was found to switch to a high-resistive state (HRS) of ∼10^8^ Ω at 1.0 V, followed by a recovery to the initial low-resistive state (LRS) reversibly with a higher applied voltage (1.3 V). Devices with larger plug sizes exhibited similar *RV* trends, indicating nonvolatile and reversible resistive switching, i.e., LRS-to-HRS (RESET process) and HRS-to-LRS (SET process). The highest HRS resistance observed was ∼10^9^ Ω, and the HRS/LRS resistance ratio was over 10^5^. Figure S5 summarizes the programming window of various NVMs including oxide ReRAM/conducting bridge RAM (CBRAM),^25,26^ nitride ReRAM,^27^ nitride FeD memory,^14^ PCM,^7^ interfacial PCM (iPCM),^7^ phase-change heterostructure (PCH),^28^ and confined PCM.^29^ Among these technologies, the nitride PCM of the current study exhibits a superior programming window comparable to CBRAM and FeD and is much larger than most PCM-based memories, suggesting good read accuracy performance and a high potential for multi-bit storage of CrN-PCM.^30^ The resistance values of both the HRS and LRS showed a strong dependence on the plug size that decreased with increasing plug size. Moreover, typical threshold switching in the HRS was observed in the same device upon application of a current sweep (pulse width: 500 μs) for a plug size of 37 × 37 nm^2^, indicating similar switching behavior to traditional chalcogenide-based PCMs. (Figure 1c) The resistance of the CrN memory cell after the threshold switching was validated through a second sweep in the same figure, which consistently remained in the LRS. This observation strongly suggests the nonvolatility of this threshold switching phenomenon. The Joule heating energy for switching operation in CrN-based memory devices was then calculated quantitatively and compared to that of a traditional PCM GST device. For all devices, the SET energy was negligible compared to the RESET energy since the resistance in the RESET state was much higher than in the SET state. To accurately assess the Joule heating energy in both GST and CrN devices, given the fluctuating resistance state during the melting process of GST under voltage pulses, we conducted *in situ* measurements of the transient current for each voltage pulse during the RESET process. As depicted in Figure 1d, the CrN device, with a plug size of 45 × 45 nm^2^, initially resided in a low-resistance SET state. A voltage pulse of 2.8 V (represented by the black dotted line plot) was applied to the memory cell. Concurrently, we measured the transient current under this pulse, as shown in the same figure. The current exhibited a relatively linear relationship with voltage during the leading-edge, indicating minimal resistance change. Subsequently, a pronounced drop in current occurred within the voltage pulse, signifying the RESET process of the CrN memory device. The transient current during the RESET process was measured to be approximately 2.8 mA. Further confirmation of the resistance in this RESET state was obtained through DC-IV measurements at a voltage of 0.1 V, revealing resistances as high as 10^8^ Ω and indicating the success of the RESET operation by application of the voltage pulse. The voltage pulse and corresponding current pulse for the RESET process in a GST device of the same size are also plotted in Figure 1d. It is obvious that the RESET current in the CrN device is smaller compared to the GST-based device. The RESET energy can be estimated by integrating the RESET voltage and current pulses over time as *E*_RESET_ = ∫ *I*_RESET_ × *V*_RESET_ × *dt*, where the RESET operation time, *t*, was defined, as shown in Figure 1d. Figure 1e shows the RESET energy as a function of the contact area of the devices; for GST memory devices, it was found to decrease with the reducing contact area, following the same scaling trend of other GST devices with the same device structure.^4,28,31^ The RESET energy of the CrN-based device was smaller than that of GST and decreased with contact size, as well. At a plug size of 37 × 37 nm^2^, the RESET energy decreased to as low as 100 pJ, representing a reduction of around one order of magnitude when compared to the same-sized GST-based device, which consumed 900 pJ. In brief, under identical programming energy conditions, nitride PCM exhibits a substantially higher on/off programming window compared to the traditional Te-based PCM and oxide-based memory technologies, as illustrated in Figure 1f. This characteristic is crucial not only for enabling low power but also for ensuring good read accuracy.

### Mechanism of Resistive Switching in CrN Device

**Figure 2:**
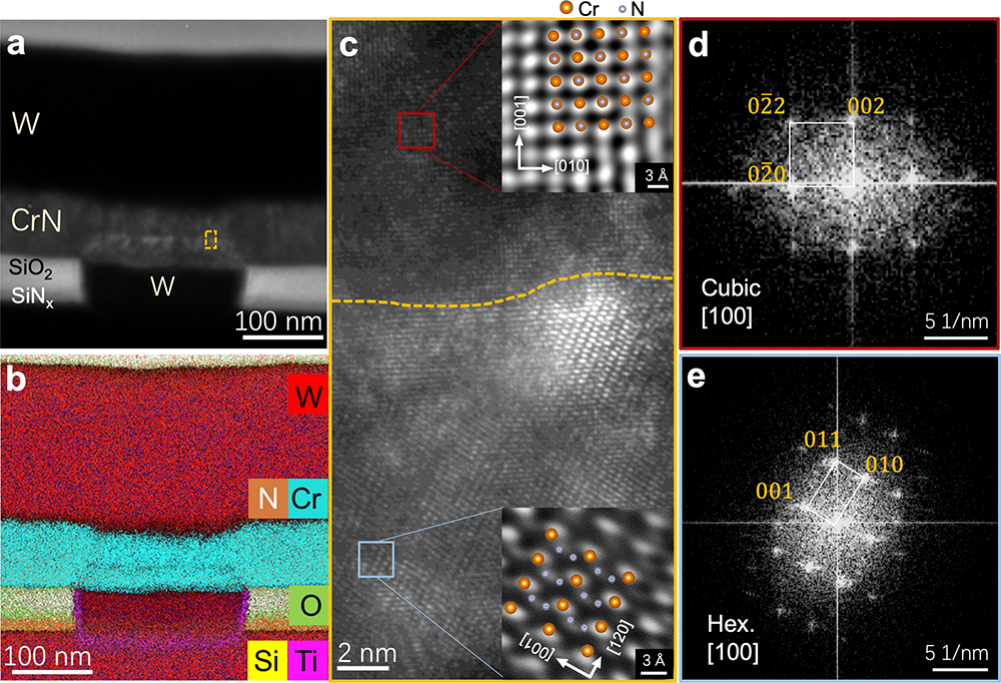
Phase-change behavior in CrN-based memory devices. (a) Cross-sectional TEM image of the device, which was previously RESET to an HRS. (b) Scanning TEM (STEM)–energy-dispersive X-ray spectroscopy elemental mapping. (c) Cross-sectional high-resolution TEM image around the boundary of the active and matrix regions, along with inverse fast Fourier transform images of the areas indicated in the red and blue boxes. (d,e) Fast Fourier transform images of the local areas from, respectively, the active and inactive regions in (c).

We conducted TEM observations of the CrN device to investigate the resistive switching mechanism. A large plug size (218 × 218 nm^2^) was used for these observations due to the limited resolution of our focused ion beam system (JEOL, JIB-4600F) during fabrication of the TEM samples. Before the TEM analysis, the device sample was switched to an HRS of ∼10^6^ Ω by the application of a voltage pulse of 3.8 V for 50 ns. A distinct bright contrast (active region) was observed in the CrN layer between the W plug and the TE electrode (Figure 2a). It should be noted that in our device substrate, an ultrathin TiN sidewall adhesion layer (3–5 nm) was used between the W plug heater and the surrounding SiO_2_ insulator region. Energy-dispersive X-ray spectroscopy (EDX) mapping of its cross-section (Figure 2b) showed the absence of obvious intermixtures among the CrN layer, W electrode layer, TiN sidewall, and insulating layer, while a Cr concentration deficit was clearly observed in the active region of the CrN layer. Figure 2c displays an atomic column image at the boundary region of the matrix (upper part) and the active region (lower part) detected by high-resolution TEM (HRTEM) in the area indicated by the yellow dotted box in Figure 2a. Figure 2d shows a fast Fourier transform (FFT) image of the matrix area indicated by the red square in Figure 2c, indicating a cubic phase. The corresponding inverse fast Fourier transform (IFFT) image of the HRTEM micrograph (square area enclosed by the red line in Figure 2c) showed a clear cubic atomic column image with a (002) plane distance of 2.05 Å, which is consistent with the lattice parameter *a* = 4.17 Å of NaCl-type cubic CrN. For the active region, instead, the FFT pattern (Figure 2e) revealed a hexagonal structure. The distance between the two Cr layers, derived from the IFFT image (bottom inset in Figure 2c), was 3.7 Å (bright spots).

Let us discuss the details of the hexagonal structure. In Cr–N binary systems, there are two hexagonal phases: Cr_2_N and CrN_2_. Since Cr_2_N is metallic,^33^ the hexagonal phase formed in the CrN layer with an HRS is unlikely to be the Cr_2_N hexagonal phase. The crystal structures of cubic CrN and hexagonal CrN_2_ are displayed in Figure S6. The IFFT image in the bottom inset in Figure 2c strongly suggests that the observed hexagonal phase is hexagonal CrN_2_ with N–N dimers present.

Both density functional theory calculations and experiments have recently revealed the formation of a CrN_2_ hexagonal phase under high pressure.^34,35^ It is also suggested theoretically that a metal-insulator transition can be induced by replacing N with a N–N dimer in WC-type metallic CrN, resulting in a semiconductor characteristic in WC-type CrN_2_.^34,35^ Zhao et al. also pointed out that the combination of N 2*p*–Cr 3*d* hybridization and electron–electron Coulomb repulsion leads to the insulating nature of CrN_2_.^35^ This reported theoretical result strongly suggests that the induced highly resistive hexagonal phase in the CrN device is CrN_2_. Moreover, the reported simulation has shown that WC-type CrN_2_ is stable with the lattice parameters *a* = *b* = 2.725 and *c* = 3.712 Å using Perdew–Burke–Ernzerhof (PBE) exchange–correlation potentials.^35^ Thus, the observed lattice parameters *a* = *b* = 2.719 and *c* = 3.712 Å of the hexagonal phase formed in the CrN layer in an HRS agree well with those of the WC-type CrN_2_ phase; the atomic column image (bottom inset in Figure 2c) can be well-matched with the atomic positions on the (110) plane of WC-type CrN_2_. The N atom position could be clearly observed in the IFFT image derived from the FFT pattern in the [121] zone axis of CrN_2_ and matched well with the WC-like hexagonal CrN_2_ structure (Figure S7).

### Nitrogen Atom Diffusion by the Soret-Effect

**Figure 3:**
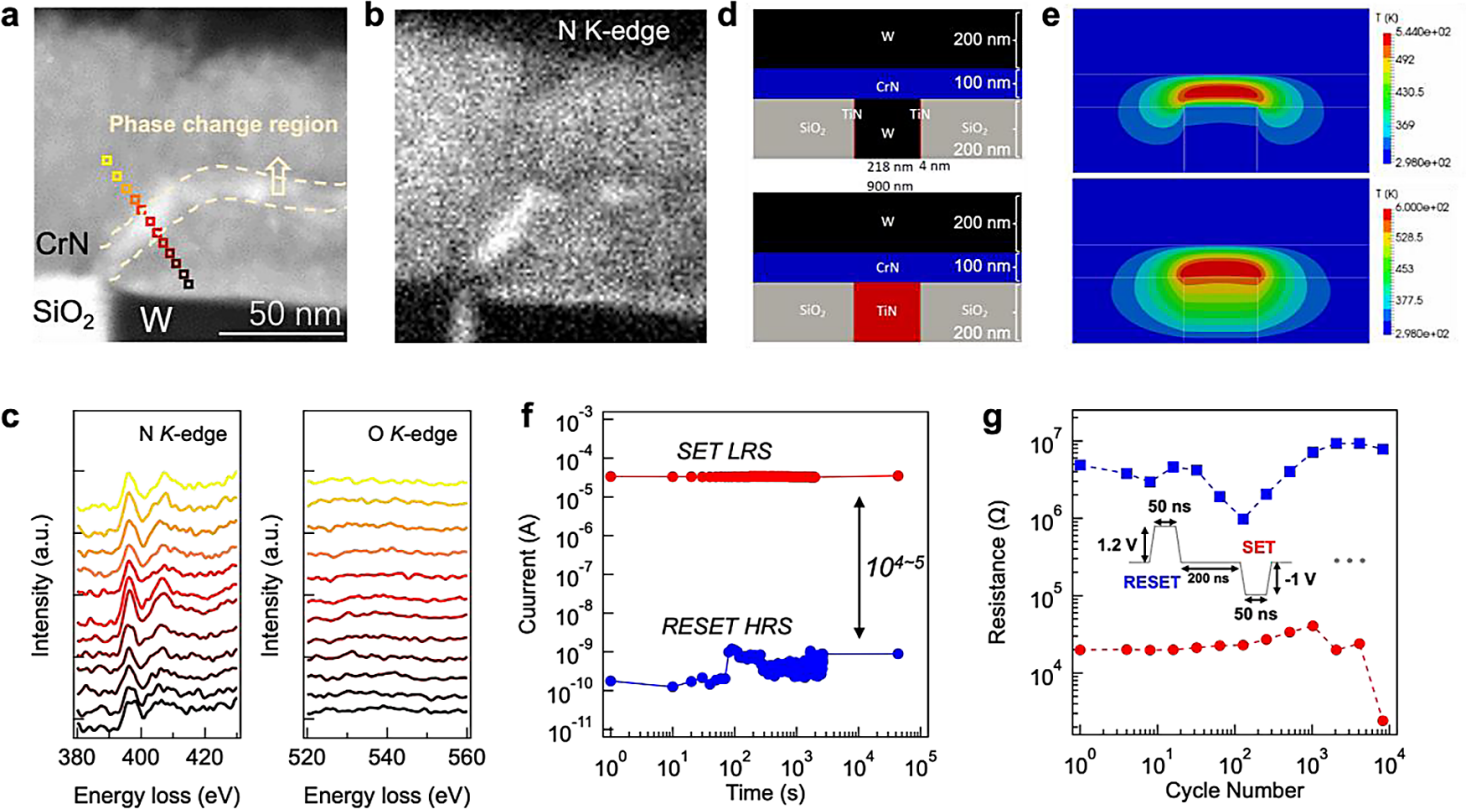
Origin of phase-change in CrN-based memory devices. (a) Cross-sectional TEM image of the CrN-based device near the phase-change region and (b) its corresponding electron energy loss spectroscopy mapping at the N *K*-edge. (c) Electron energy loss spectroscopy (EELS) of the N *K*-edge (left) and the O *K*-edge (right) taken from the marked points in (a). (d) Cross-sectional schematic of the CrN-based device for thermal distribution simulation and (e) simulated results for a W plug with a 4 nm TiN adhesion layer (upper) and a TiN plug (bottom) for comparison. (f) Current of HRS and LRS of the CrN-based memory cell under the voltage of 0.1 V as a function of measurement time at room temperature. (g) Results of endurance tests on cells with a plug size of 45 × 45 nm^2^.

To induce the hexagonal CrN_2_ phase formation in the CrN matrix, the composition must change simultaneously with the crystal structure, which is a different process from conventional PCMs that undergo amorphization/crystallization without overall composition change. Thus, Cr or N atoms must diffuse during the formation of N–N dimers upon phase change in the CrN layer. As confirmed by EDX in Figure 2b, a poor Cr concentration was clearly observed in the active region of the CrN layer, indicating the diffusion of Cr atoms during phase change. However, it is difficult to accurately detect a light element such as N using EDX. Therefore, electron energy loss spectroscopy (EELS) which exhibits high sensitivity for light atoms such as N was employed. Figure 3a,b shows the TEM image and EELS mapping of the CrN layer near the active region, obtained in the energy range of 408–422 eV. The N *K*-edge mapping clearly indicates a higher N concentration in the active region than in the CrN matrix. Hence, the combination of this EELS analysis with the EDX results (Figure 2b) confirms Cr-poor and N-rich compositions in the active region. The N in the TiN side wall can also be clearly observed from EELS N mapping (Figure 3b). As shown in Figure 3a, the phase-change active region in the CrN layer was just above the TiN thin layer rather than on the entire surface of the W plug as usually observed in the conventional PCRAM.^7^ While the local composition variation induced by electrical pulses bears some resemblance to the filament formation mechanism observed in ReRAM, the CrN phase-change process between the LRS and HRS aligns more closely with the switching mechanism observed in traditional phase-change materials for PCRAM, where a high-resistance amorphous volume forms within a conductive crystalline matrix through Joule heating. We also obtained an EELS line profile of the O *K*-edge (Figure 3a,c) across the phase-change region to investigate the change in O dopant distribution upon the phase change, where an EELS line profile of the N *K*-edge was also obtained for comparison. The peak intensity of O was very small, and no significant O composition was observed upon the phase-change. To clearly observe the peak shape of O *K*-edge, we obtained EELS data taken from a wider area in the phase-change region and matrix region, as shown in Figure S8a. A stronger peak intensity of O *K*-edge can be observed in Figure S8b. In both spectra, a pre-edge of the O *K*-edge is evident, suggesting the splitting of the Cr 3*d* orbitals in a six-coordinated environment. The smaller intensity of the pre-edge in the phase-change region may be attributed to the local coordination changes tuned by O. To confirm the effect of O on the phase-change mechanism, we also tried to fabricate a pure CrN film (designated as CrN′ hereafter). The vacuum condition of a sputtering chamber and sputtering condition to obtain a pure CrN film without O contamination are very strict, which has become a remained open question in many fields such as coating and thermoelectric applications.^36−38^ Here, we found that the O content could be decreased by lowering the working pressure of the sputtering chamber (Supporting Information 9). Based on the results, we successfully obtained an O-free CrN′ film exhibiting the same crystal structure (NaCl-cubic) with the CrN film (Figure S10), where the cation/anion composition ratio was 1.02 (Supporting Information 12). The CrN′ memory device exhibits similar *RV* characteristics, and the TEM observation after the RESET operation reveals that the CrN′ device undergoes the phase-change from NaCl-type cubic CrN to WC-type hexagonal CrN_2_, implying that this phase-change is an intrinsic behavior of CrN (Supporting Information 11). In addition, interestingly enough, a different carrier type was detected in CrN (*p*-type) and CrN′ (*n*-type) thin films by both Seebeck and Hall measurements (Figure S12). Le Febvrier et al. demonstrated that p-type conduction can be attributed to Cr vacancies, which push the Fermi level down toward the valence band.^39^ We also directly observed the chemical environment around Cr atoms in a CrN thin film using the Cr K-edge by extended X-ray absorption fine structure (EXAFS) and found that the coordination number of Cr–Cr was far from the theoretical value of 12, indicating a Cr nonstoichiometric deficiency. (See experimental conditions and fitting results of EXAFS in Supporting Information 13.) Therefore, it is surmised that the *p*-type conduction is likely due to holes generated by Cr vacancies, while the *n*-type conduction in CrN′ is commonly attributed to N vacancies serving as electron donors.^39^ The band structures of both CrN and CrN′ were measured to determine their respective *p*-type and *n*-type conduction properties, as evidenced by their optical bandgap and Fermi levels determined with respect to the valence band maximum by hard X-ray photoelectron spectroscopy (HAXPES) measurements (Supporting Information 14). The same switching behavior between the CrN- and CrN′-based devices strongly supports that the defects or the carrier type have a minor effect on the switching properties of a CrN film. Hence, while unintentional O incorporation and the associated change in cation/anion composition ratio have a significant impact on the electrical characteristics of CrN, it exerts a minimal influence on the switching mechanism based on the phase change between cubic CrN and hexagonal CrN_2_. Nonetheless, it is worth noting that the dopant, like in many PCMs, may still affect kinetic processes or switching speeds due to potential changes in the local structure.^40^

Thus, to understand the driving force of nitrogen atom diffusion and phase change in the CrN devices, we simulated the thermal distribution under the application of a voltage.^41^ In this simulation, thermoelectric effects such as the Thomson effect, Peltier effect, and thermal boundary resistances were ignored as well as the contact resistance between the low-resistive CrN (semimetal-like) and metal electrodes because of its minor effect on device operation. The specific structure and size of the simulated CrN-based memory device is shown in Figure 3d. To understand the effect of the TiN adhesion layer between the W plug electrode and SiO_2_ insulator on the thermal distribution when voltage was applied, a single TiN plug-type memory device was also simulated. A positive voltage input of 0.2 V to the BE was simulated, along with Joule heating generated within the memory structure (Supporting Information 15). For the CrN-based memory device, the simulated temperature distribution (Figure 3e) suggested that when a TiN adhesion layer exists between the W plug and SiO_2_ insulator, the hottest region inside the CrN layer is circularly distributed in an arc from near the top of the TiN sidewall layer to the middle of the CrN layer. This hottest region agrees well with the active region showing phase-change in the CrN layer, which strongly supports the supposition that Joule heating is a key in the phase-change phenomenon. For the single TiN plug heater, instead, the hottest region covers the entire interface between the TiN and the CrN layers. In this case, phase change cannot occur just above the W plug heater because the Joule heating dissipates easily toward the W heater due to the higher thermal conductivity of W compared with TiN, resulting in less residual heat above the heater.^42^ We also simulated the electrical field distribution under the application of a voltage in a CrN device with a W plug/TiN sidewall heater or a TiN plug heater (Figure S15). The electric field was concentrated near the surface of the W plug heater rather than the inside of the CrN layer due to the highly conductive W, similar to electric field distributions in ReRAM devices.^43^ These results further indicate that atomic diffusion in the central region of the CrN layer and phase change are driven by the thermal effects rather than the electrical field effects. An established thermophoresis/diffusion mechanism, the so-called Soret–Fick diffusion of atoms or vacancies, can well explain the nonelectrical field dependency of N atoms in this case.^44^ A large radial temperature gradient is generated, and the Soret-effect can become the dominant factor influencing atomic migration; the sign of the applied electrical field and the carrier types in CrN are not important for the Soret-effect.^45^ It is noteworthy that phase change after the above thermal diffusion process can be stable and nonvolatile at room temperature, as indicated in current-time measurements, which show only a minor drift. (Figure 3f) To further confirm the phase stability of CrN_2_ in the memory cell, we conducted temperature-dependent resistance change (*R*–*T*) measurements on our memory device. However, due to the small pad size of the T-shape device compared with our experimental probes, we fabricated a device with larger electrode pads. The memory cell was initially in the low-resistance SET state and was RESET to an HRS using voltage pulses. (Figure S16a) The HRS memory cell was then transferred to a probe furnace and annealed under an Ar atmosphere with a heating rate of 10 °C/min up to 400 °C. The *R*–*T* curve of the HRS CrN memory cells is shown in Figure S16b,c. The resistance decreased slightly with increasing temperature until the phase change point (*T*_phase change_) was reached, after which the resistance decreased sharply corresponding to a phase change from CrN_2_ to CrN. The *T*_phase change_ was then determined to lie within the range of 250–300 °C by observation of the minimum of the first derivative of the *R*–*T* curve, which yielded a value much higher than the crystallization temperature of traditional PCM: GST (∼150 °C).^46^ Cyclic resistive switching of the CrN device was also tested to over 10^4^ cycles, which is still limited compared to other PCMs such as GST (10^5–12^ cycles). (Figure 3g) We observed a strong correlation between endurance and phase-change volume, which can be, in turn, controlled by adjusting the amplitude of the applied voltage pulses. A larger phase-change volume leads to more severe atomic diffusion, making the device more prone to failure. (Supporting Information 17) Therefore, it is speculated that device endurance can be enhanced by implementing a confined PCRAM device structure to restrict the extent of atomic migration.^29^ In short, CrN memory exhibits a nonvolatile and reversible phase-change property with overall-qualified switching performance by comparison with not only the traditional GST but also the other melting-free PCMs such as MnTe. (Table S3)

**Figure 4:**
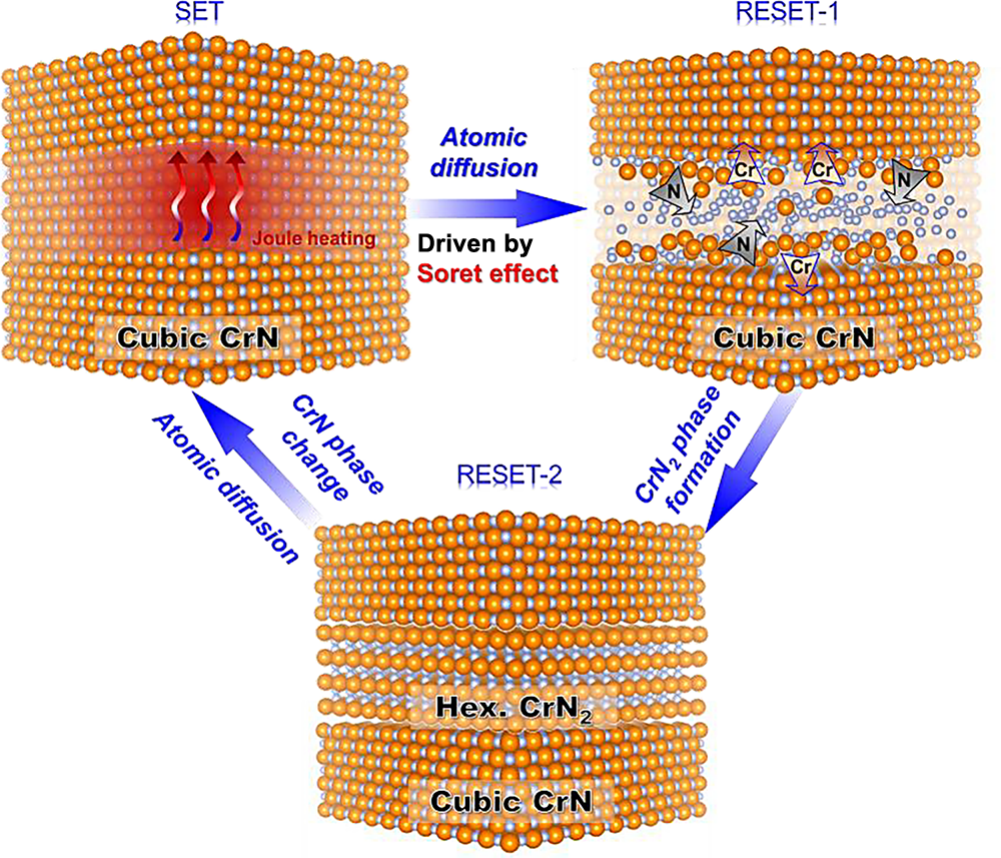
Diagram of the resistive switching mechanism of phase-change CrN.

Here, the switching mechanism of phase-change CrN can be summarized in Figure 4. The SET state originally shows the low resistance of a cubic CrN phase. It has been reported that hexagonal CrN_2_ can be synthesized through a direct chemical reaction between chromium and molecular nitrogen at high temperature and pressure.^34^ In our device, when subjected to an external heat stimulus by an electrical pulse, N atoms gather in a localized high-temperature region via the Soret-effect, which stabilizes the hexagonal CrN_2_ at the hottest region. (RESET-1 state) At the same time, the hottest region is constraint from the surrounding matrix, which may also be a factor to stabilize the hexagonal CrN_2_. During the falling process of the pulse, the heat source is reduced and removed on the order of a nanosecond, and therefore, the hexagonal CrN_2_ phase formed in the hottest region is frozen to room temperature. This rapid cooling process allows the formation of the metastable CrN_2_ phase at room temperature (quenchable), preventing atoms from diffusing and reverting to the stable cubic phase once more. (RESET-2 state) The two-step RESET process in the CrN memory device is also evident from the transient current change under an applied voltage pulse. Initially, the CrN memory device was in a low-resistance SET state. We applied a voltage pulse with a pulse width of 1 μs and an amplitude of 1.6 V. The leading width was set to 1 μs with a step of 20 ns. The current was measured *in situ* with the applied voltage (Figure S18a). The current increased with voltage and exhibited a slight decrease when the voltage reached 1.6 V. This observation suggests a slight change in the resistive state of the memory cell, which could be attributed to a first-stage diffusion process driven by the Soret-effect (RESET-1). Subsequently, the current abruptly dropped while maintaining 1.6 V, indicating the initiation of the phase-change to CrN_2_. Following the cooling process of RESET-2, the current could no longer be detected within the same current range (Figure S18b), indicating the formation of a high-resistance CrN_2_ region within the memory cell. To verify the reversibility of the atomic diffusion process, we examined the LRS memory cell by TEM. The cross-sectional TEM image of the CrN memory cell (218 × 218 nm^2^) after a SET operation from HRS (4.5 V, 50 ns) is shown in Figure S19a. From the EELS and EDX mapping, we observed that the N-rich and Cr-poor region within the phase-change area nearly vanished compared to the RESET state, implying that by Joule heating, the diffusion of N atoms occurs to form a stable CrN phase. (Figure S19b,c)

The above process is generally different from the conventional amorphous–crystalline chalcogenides-based PCMs. The diffusion behavior of atoms prior to the phase change in the CrN memory device exhibits some similarity to the forming process of conductive filament paths in oxide-based ReRAM, which is reversible and fast.^47^ However, the matrix of the oxide layer in ReRAM is typically insulating, and the filament phase is often chemically and thermally unstable, leading to poor data retention properties.^48,49^ In contrast, the CrN memory device in this study combines the benefits of phase change in PCRAM and the thermally induced atomic diffusion process in ReRAM, providing a promising approach to addressing the challenges of the high-energy-budget melting process in PCRAM and the poor thermal stability of ReRAM simultaneously.

## Conclusions

In summary, we report a memory device based on the fast and reversible phase-change of a simple toxic element free and easily fabricated CrN thin film. Phase change CrN exhibits unipolar resistive switching properties and requires a lower operation energy than traditional GST-based PCMs. Our experiments revealed that the nonvolatile resistive switching mechanism originates from the phase change between the low-resistivity NaCl-type cubic CrN phase and the highly resistive WC-type hexagonal CrN_2_ phase, as induced by the Soret-effect. The CrN device exhibits a large ON/OFF ratio (more than 10^5^) and fast switching speeds (∼30 ns) while requiring low operating energy (∼100 pJ), resulting in superior performance to a typical GST-based memory. This study provides not only possibilities for the application of crystalline-to-crystalline PCMs as next-generation NVM devices but also a further investigation of low-cost, clean, and chalcogenide-alternative PCMs, which is compatible with a CMOS integration circuit. Furthermore, the same crystal structure of the *n*-type and *p*-type CrN films obtained by tuning O content indicates a great potential in the homogeneous *pn* junction to serve as a diode-type selector element in a memory device.^50−52^

## Experimental Section

### Preparation of CrN Thin Films

CrN films were deposited on SiO_2_ (100 nm)/Si (725 um) or glass (Corning EAGLE XG) substrates by the radiofrequency (RF) magnetron reactive sputtering of Cr (99.99%) pure targets at room temperature in an Ar/N_2_ (5:3) gas atmosphere. where the substrate holder was rotated during deposition; the RF power was fixed to 50 W, the base pressure of the sputtering chamber was below 5.0 × 10^–5^ Pa, and the working pressure was ∼4.4 × 10^–1^ Pa. The film thickness was confirmed with an atomic force microscope (Keyence, VN-8000).

### Characterization of CrN Thin Films

The composition of 30 nm-thick CrN thin films deposited on a SiO_2_ (100 nm)/Si (725 um) substrate was assessed by RBS (National Electrostatics Corp., Pelletron 3SDH). The crystal structures were investigated by XRD (Rigaku, Ultima IV); the diffraction patterns were taken in the 2θ range from 35° to 50° with Cu Kα radiation. The cross-sectional microstructures were observed by using a TEM system (JEOL, JEM-2100F) at an accelerating voltage of 200 kV. For this analysis, the samples were thinned via an ion milling instrument (Gatan, PIPS). The samples for the XRD and TEM measurements were deposited on a SiO_2_ (100 nm)/Si (725 um) substrate with a thickness of ∼100 nm.

The electrical properties were evaluated through a Hall effect measurement apparatus (Toyo Corp., ResiTest 8400). The Seebeck coefficient was measured with a ResiTest 8300 (Toyo Corporation) in the temperature range of 260–400 K. The samples for the Hall effect and Seebeck coefficient measurements were grown on glass substrates with a thickness of 100 nm.

### Memory Device Fabrication

T-shaped memory devices with W electrode plugs were used. The device size was initially defined by the size of the plug-type bottom W electrode, which was 34 to 218 nm in the side length (Figure S1). The substrate wafer with the W plug was fabricated using standard semiconductor processes: photolithography, etching, metal filling, and chemical–mechanical polishing (CMP). After the wafer was cut into small substrates, the substrates were first etched for 73 min by an Ar plasma to remove the surface oxidation of the W plugs. A 100 nm CrN or CrN′ layer was *in situ* deposited on top of the W plug via conventional lithography. Then, a 250 nm W TE was deposited onto it.

### Memory Device Characterization

The read resistance and DC current–voltage sweep for all devices were measured by using a semiconductor parameter analyzer (Keysight, B1500A). To evaluate the resistive switching properties, pulse generators (Keysight, B1525A) were used to apply short voltage pulses to the memory cells; the pulse amplitude and width were confirmed by an oscilloscope (Tektronix, TBS 1202B). The transient current under the voltage pulse was monitored by a waveform generator/fast measurement unit (WGFMU) integrated into the B1500A (Keysight, B1530A).

The cross-sectional microstructure of the devices after the RESET operation (i.e., from LRS-to-HRS) was observed with a TEM instrument (JEOL, JEM-2100F) at an accelerating voltage of 200 kV. An EDX system (JEOL, JEM-2100F) was utilized to map the elemental distributions in the T-shaped device. To obtain atomic images, an HRTEM apparatus equipped with an ADF-STEM detector (JEOL, ARM200F) was used. IFFT images were obtained by using the Gatan DigitalMicrograph software. An EELS spectroscope (JEOL, ARM200F) was utilized to identify the concentration of N atoms in the cross-section of the devices. The TEM samples of the device cross sections were thinned by a focused ion beam system (JEOL, JIB-4600F) with a Ga ion beam at 30 keV and polished at 10 keV.

## Data Availability

The data are available from the corresponding authors upon request.

## Supporting Information Available

Fabrication of the CrN-based memory device; crystal structure of the CrN thin film; temperature dependence of resistivity of CrN and CrN′ thin films; setup for measuring the switching properties; programming window comparison of various NVMs; crystal structures of Cr–N compounds; N sites in hexagonal CrN_2_; EELS spectra of O *K*-edge; oxygen content as a function of working pressure; crystal structure of the CrN′ thin film; switching performance and phase change behavior of CrN′ memory; carrier types in CrN and CrN′ thin films; EXAFS measurements on CrN; band structure of CrN and CrN′; thermal distribution simulation in CrN memory cell; thermal stability of CrN_2_ phase; cyclic resistive switching of CrN memory; transient current measurement in CrN memory device; and TEM observation in Set state of CrN memory device (PDF)

## Notes

Yi Shuang, Shunsuke Mori, Takuya Yamamoto, Shogo, Hatayama, Yun-Heub Song, Jin-Pyo Hong, Daisuke Ando, Yuji Sutou. Soret-effect induced phase-change in chromium nitride semiconductor film. *Research Square Preprint*, 2022. DOI: 10.21203/rs.3.rs-2486043/v1. Posted Date: February 1st, 2023
