# Supplementary material for: Soret-Effect Induced Phase-Change in a Chromium Nitride Semiconductor Film
Source: ACS Nano. 2024 Aug 1;18(32):21135–43. doi: 10.1021/acsnano.4c03574 (PMC11328172; doi:10.1021/acsnano.4c03574)
Supplement: Supplementary file 1 — nn4c03574_si_001.pdf [file nn4c03574_si_001.pdf]

Supporting Information

Soret-effect induced phase-change in a chromium nitride semiconductor film

Yi Shuang[1], Shunsuke Mori[2], Takuya Yamamoto[3], Shogo Hatayama[2], Yuta Saito[4], Paul J. Fons[5], Yun-Heub Song[6], Jin-Pyo Hong[7], Daisuke Ando[2], Yuji Sutou[1,2]**

[1]WPI Advanced Institute for Materials Research, Tohoku University, 2-1-1 Katahira, Aoba, Sendai 980-8577, Japan

[2]Department of Materials Science, Graduate School of Engineering, Tohoku University, 6-6-11 Aoba-yama, Sendai 980-8579, Japan

[3]Department of Metallurgy, Graduate School of Engineering, Tohoku University, Miyagi 980-8579, Japan

[4]Device Technology Research Institute, National Institute of Advanced Industrial Science and Technology (AIST), Tsukuba Central 2, Umezono 1-1-1, Tsukuba 305-8568, Japan

[5]Department of Electronics and Electrical Engineering, Faculty of Science and Technology, Keio University, 3-14-1 Hiyoshi, Kohoku-ku, Yokohama, Kanagawa 223- 8522, Japan

[6]Department of Electronic Engineering, Hanyang University, 17 Haengdang-dong, Seongdong-gu, Seoul 133-791, Korea

[7]Department of Physics, Hanyang University, Seoul, 04763, Korea

*Correspondence to:
shuang.yi.e3@tohoku.ac.jp (Y. Shuang), ysutou@material.tohoku.ac.jp (Y. Sutou)

1. Fabrication of the CrN-based memory device.

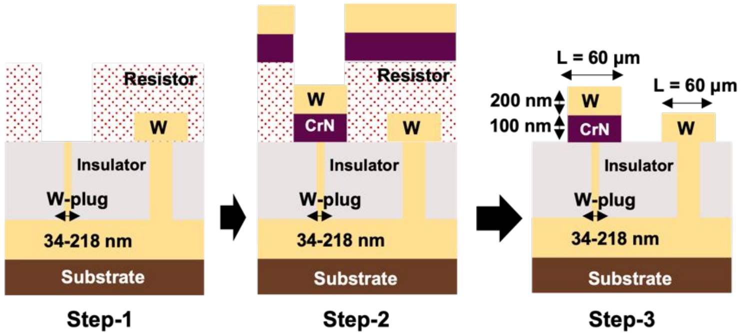

Fig. S1. Process flow to fabricate CrN layer and top electrode: T-shaped memory cell substrates cut from substrate wafer with the W-plug electrode were used in this study. It consisted of a square W plug (heater electrode) with a side length varying from 34 to 218 nm. The fabrication process was initiated from patterning and photolithography. (Step-1) In this Step-1, a negative photoresist (ZPN1150-90, ZEON, Japan) was spin-coated onto the substrate using a spin-coater (ACT-300A, ACTIVE) at a speed of 4000 rpm and then baked at 105 ºC for 120 s after coating. The device was then patterned using a photolithography machine (PEM-800, Union Optical Co., Ltd.). The second bake process was carried out at 115 ºC for 60 s immediately after photolithography. The device was developed in the developer (NMD-3, TOKYO OHKA KOGYO CO., LTD.) for 70 s and cleaned with distilled water for 50 s. The remaining resistor in the patterned area was cleaned using a UV irradiation chamber for 180 s. In Step-2, the device was first reverse sputtered for 73 min to remove the surface oxidation of W-plug and the CrN or CrN’ layer (100 nm) and the W top electrode layer (200 nm) were sequentially deposited onto the patterned area after photolithography at room temperature by sputtering. In Step-3, a lift-off process was conducted by immersing the device in acetone solvent at room temperature until all the W/CrN/resist layer was removed. The device was finally cleaned with ethanol (60 s), distilled water (60 s), dried and wait for test. The CrN device was fabricated with the plug’s side length of 37, 45, 81, 113 and 218 nm and the CrN’ device with the size of 34, 37, and 45 nm.

2. Crystal structure of the CrN thin film.

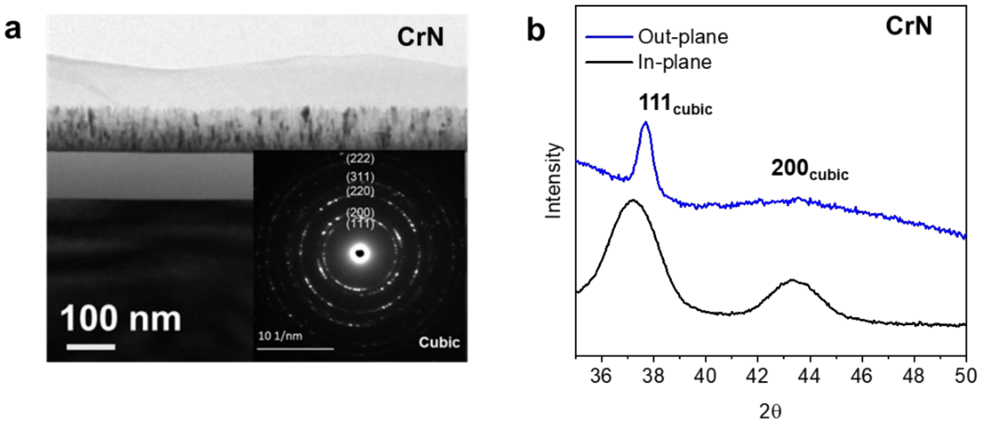

Fig. S2. (**a**) Bright-field cross-section TEM micrograph, revealing a columnar-like grain microstructure and a relatively smooth film surface; the inset displays a selected area electron diffraction (SAED) pattern of the film, showing a ring pattern of the polycrystalline phase that can be attributed to a single NaCl-like cubic CrN phase. (**b**) In- and out-of-plane XRD spectra at room temperature, indicating a cubic phase.

3. Temperature dependence of resistivity.

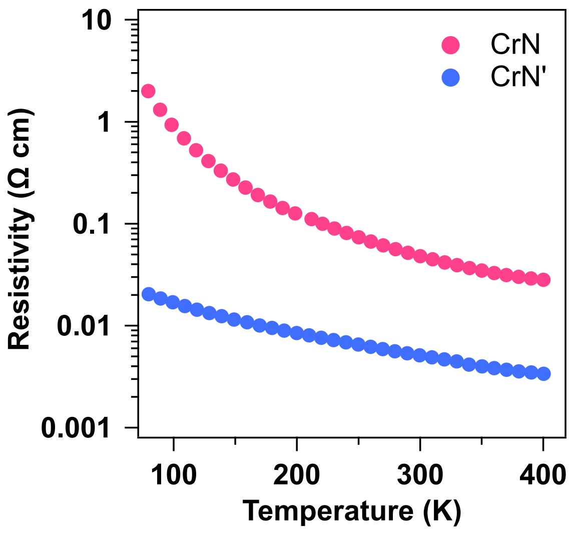

Fig. S3. The resistivity of CrN and CrN’ measured from Hall measurements at various temperatures from 80 to 400 K.

4. Setup for measuring the switching properties.

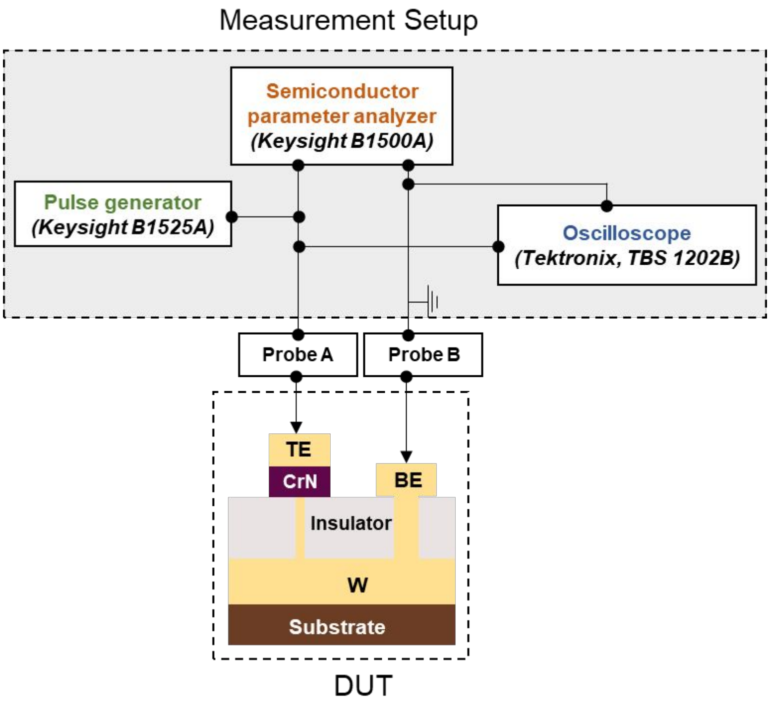

Fig. S4. The DC read resistance of the device was measured using a semiconductor parameter analyzer. To evaluate the resistive switching properties, a pulse generator was used to apply short voltage pulses to the device under test (DUT), and the pulse amplitude and pulse were confirmed by an oscilloscope.

5. Programming window comparison of various NVMs

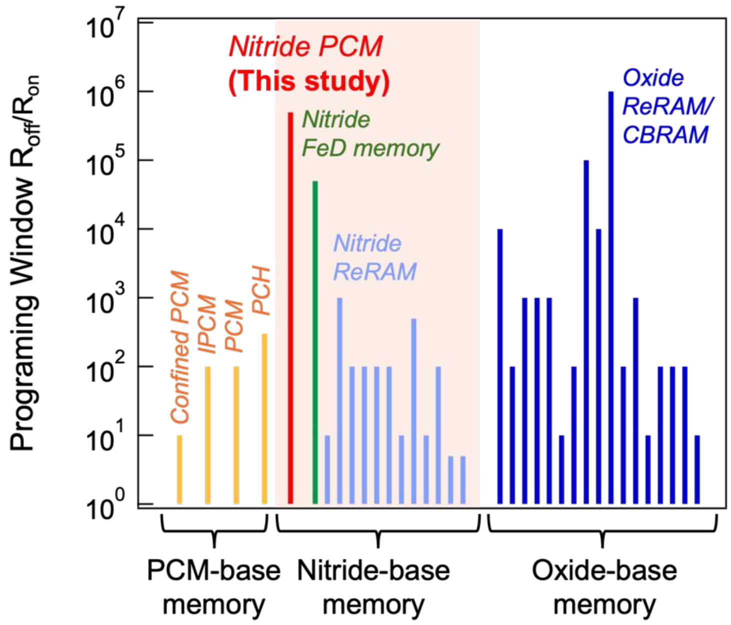

Fig. S5. Programming window of various next-generation nonvolatile memories [1–6].

6. Crystal structures of Cr-N compounds.

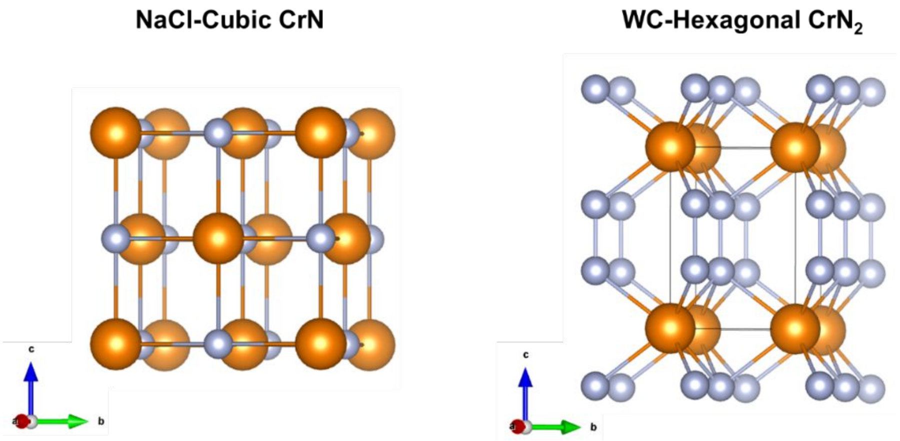

Fig. S6. Crystal structures of NaCl-like cubic CrN and WC-like hexagonal CrN_2. By calculating the densities of these two structures, a density of 6.0 g/cm^3 for cubic CrN and 5.7 g/cm^3 for hexagonal CrN_2 phase was obtained. The observed density change is approximately 5%, a value comparable to that typically observed in traditional PCMs.

7. N sites in hexagonal CrN_2.

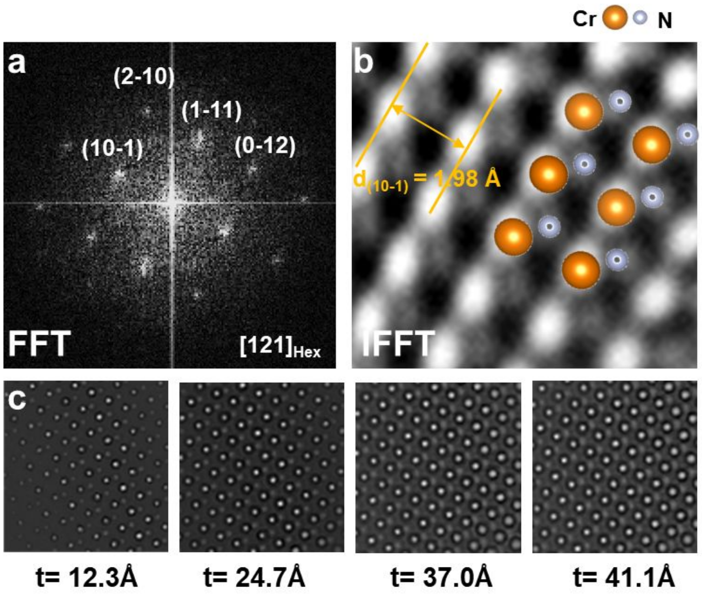

Fig. S7. (**a**) FFT and (**b**) IFFT image derived from the [121] zone axis, indicating the location of N atoms. (**c**) Simulated thickness/defocus map of (**b**) obtained by the multislice method from HRTEM images by using the QSTEM software [7]; for the simulation, the lattice parameters a = 2.71931 Å and c = 3.71155 Å were used for hexagonal CrN_2. The IFFT images were obtained using the Gatan DigitalMicrograph software.

8. EELS spectra of O *K*-edge.

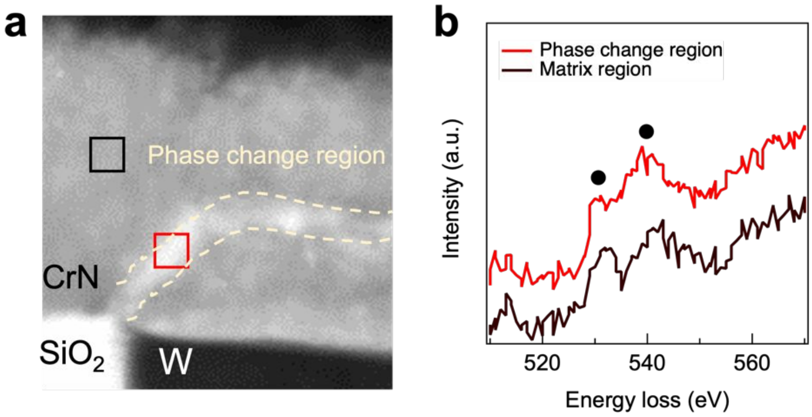

Fig. S8. (**a**) Cross-sectional TEM image of a CrN-based device near the phase-change region. (**b**) The electron energy loss spectroscopy (EELS) of the O *K*-edge taken from the marked points in (**a**)

9. Oxygen content as function of working pressure.

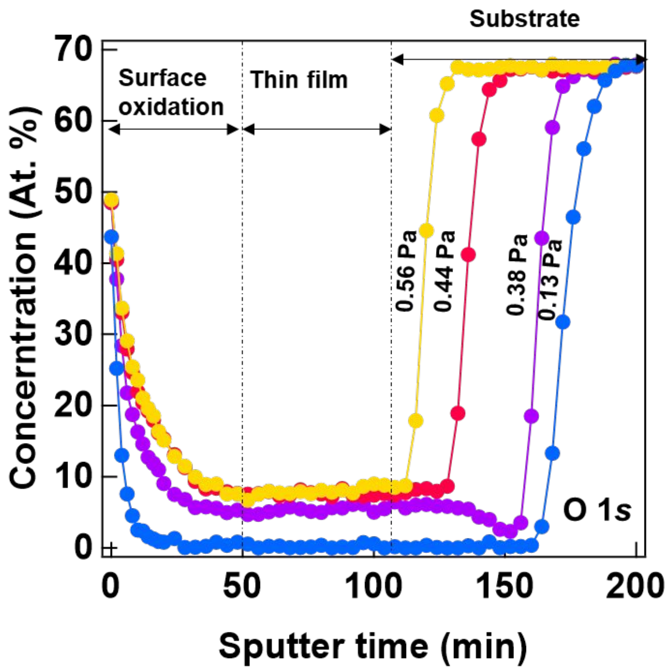

Fig. S9. Oxygen concentration profiles of CrN films sputtered at various working pressures obtained by X-ray Photoelectron Spectroscopy (XPS, Quantum 2000, ULVAC-PHI). It can be clearly seen that the O content in the flat region of the thin-film profile decreases with decreasing working pressure, with O content reaching almost 0 at the working pressure 0.13 Pa.

10. Crystal structure of the CrN' thin film.

The CrN' film was deposited using the same sputter chamber and conditions except for the working pressure, which was 1.3×10^{-1} Pa. The TEM and XRD of CrN' film were measured using same method with CrN thin film.

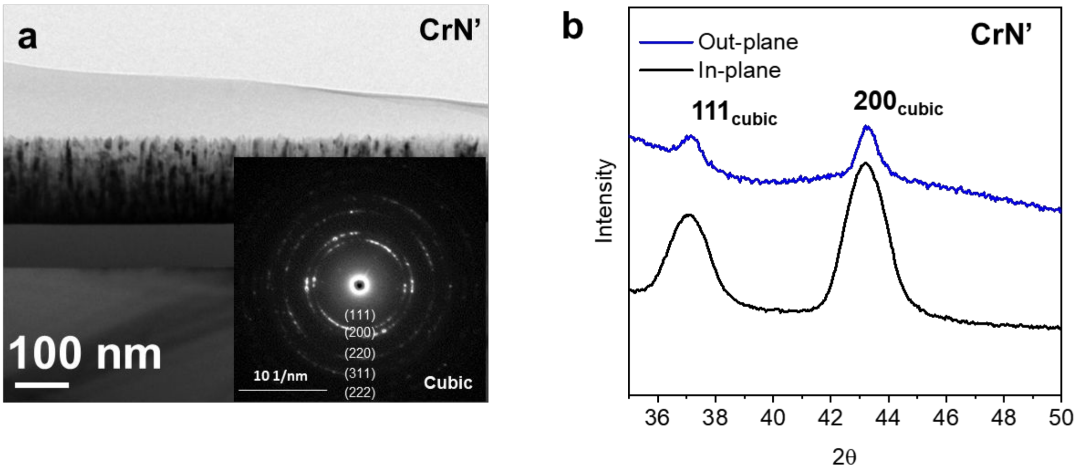

Fig. S10. (**a**) Bright-field cross-section TEM micrograph, exhibiting a microstructure similar to the CrN thin film shown in **Fig. S2a**, along with a SAED pattern (inset) showing a ring pattern of the polycrystalline phase, which can be indexed as a single NaCl-like cubic CrN phase. (**b**) In- and out-of-plane XRD spectra at room temperature, indicating a cubic phase.

11. The switching performance and phase change behavior of CrN’ memory.

We tested the switching performance of the CrN’-based memory device under electrical pulses. **Fig. S11a** displays the resulting *RV* characteristics. For a device with a plug size of 34 nm × 34 nm, when we applied a positive voltage pulse to the bottom electrode (BE), the device reached the HRS of ~10^8 **Ω** at 1.2 V and then, recovered its initial LRS reversibly upon application of a higher voltage (1.5 V). Devices with larger plugs exhibited similar *RV* trends, indicating nonvolatile and reversible resistive switching, i.e., LRS-to-HRS (RESET process) and HRS-to-LRS (SET process). The highest HRS resistance observed was ~10^9 Ω, and the HRS/LRS resistance ratio was as high as 10^6. When a positive-current pulse sweep (pulse width: 500 μS) is applied to the HRS, typical threshold switching was observed, similar to that observed in a CrN memory cell. (**Fig. S11b)**

TEM observations were conducted to investigate the resistive switching mechanism of the device with a plug size of 218 nm × 218 nm. Before the TEM analysis, the device sample was switched to an HRS of ~10^6 Ω by applying a voltage pulse of 5 V for 50 ns. A distinct bright contrast was observed in the CrN’ layer between the W plug and TE electrode (**Fig. S11c**). **Fig. S11d** displays a high-resolution TEM (HRTEM) image taken at the boundary region between the matrix (upper part) and active region (lower part) indicated by a light-blue square in **Fig. S11d**. **Fig. S11e** illustrates an inverse fast Fourier transform (IFFT) image of the boundary region between matrix and active areas that is enclosed by a light-blue square in **Fig. S11d**; the bright and darker spots were supposed to be Cr and N atoms, respectively. On the matrix side, the IFFT image shows a clear cubic atomic column structure of CrN’, which is consistent with the lattice parameter a = 4.17 Å of a NaCl-like cubic structure. For the active region, the IFFT image reveals a new atomic column structure that can be indexed not as cubic but as hexagonal, indicating a phase change from cubic to a hexagonal structure.

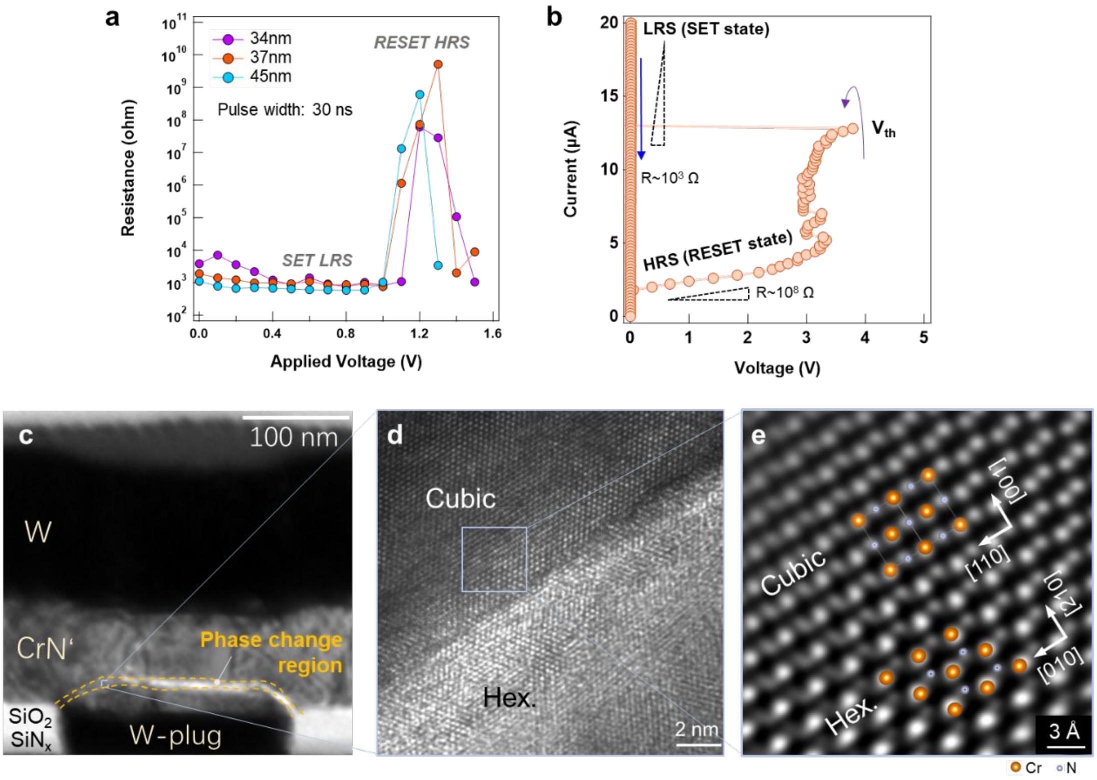

Fig. S11. (**a**) Resistance as a function of the pulse voltage for various plug sizes; the pulse width was fixed at 30 ns and the read voltage was 0.1 V. (**b**) Threshold switching behavior, showing a resistance change from ~10^8 (high-resistance state: HRS) to ~10^3 (low-resistance state: LRS). (**c**) Cross-sectional transmission electron microscopy (TEM) image of the device with a 218 nm × 218 nm plug, which was previously reset to a high-resistance state. (**d**) Cross-sectional high-resolution TEM image taken at the boundary of the phase-change region in (**c**). (**e**) Inverse fast Fourier transform image of local areas from the active and inactive region in (**d**).

12. The carrier types in CrN and CrN' thin films.

Febvrier et al. recently obtained *p*-type CrN by controlling the stoichiometry without the introduction of additional dopants using DC magnetron sputtering; they demonstrated that the *p*-type conduction can be attributed to Cr vacancies, which push the Fermi level down toward the valence band.[8] In the present study, we first obtained a CrN film with a composition of Cr 47.5 at%, N 43.9 at%, and O 8.6 at%, which was detected by RBS, where the Cr/(N+O) composition ratio was 0.91. A small amount of oxygen was introduced in the case of sputtering deposition at a high working pressure. In this case, the total concentration of N and O anions exceeded that of Cr, indicating a Cr-deficient composition and, thus, resulting in *p*-type conduction by Cr vacancies, as shown in **Fig. S12** (upper figure). By lowering the working pressure during sputtering deposition, we obtained an O-free CrN film (designated as CrN') with a Cr/N composition ratio of 1.02. This N-deficient composition film with a NaCl-type cubic structure showed *n*-type semiconductor behavior, as shown in **Fig. S12** (bottom figure).

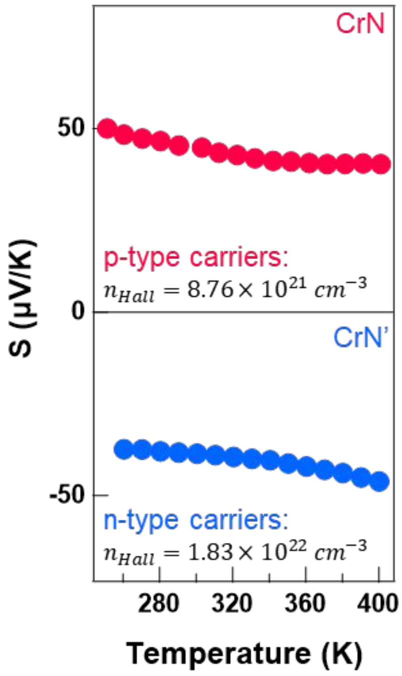

Fig. S12. Seebeck coefficients of CrN and CrN' at various temperatures.

13. EXAFS measurements on CrN

We directly observed the chemical environment of Cr k-edge in our CrN thin film by Extended X-ray Absorption Fine Structure (EXAFS, beamline BL01B1@SPring-8). In this observation, the Si (111) and Si (311) settings of the double-crystal monochromators were used. 1.3-μm-thick CrN films at N_2 flow rate of 6 SCCM were deposited onto both sides of aluminum foil for the measurements. A Si-N protection layer of 50 nm was in-situ deposited in the same sputter chamber to avoid the unwanted surface oxidation. The obtained EXAFS data were analyzed using Athena and Artemis software. [9] Fourier transform (FT) of k^3-weighted χ (k) spectra vs. radial distance (R) are shown in **Fig. S13**. Since that there is no other phase separated other than cubic-CrN phase in the film and the χ(k)-R spectrum was found to be deviated from the chromium oxide, we consider the first shell of theoretical cubic-CrN consisting of Cr bonded with 6 N atoms ($R_{Cr\text{-}N}$= 2.08 Å) and 12 Cr atoms ($R_{Cr\text{-}Cr}$= 2.95 Å).[10] The best fit was done for this plot within the *k*-range 3-16 Å and the fitting was performed in R-space range of 1-2.8 Å. (**Fig. S13**) The corresponding fitting parameters are summarized in **Table S1**. In the CrN thin film, the fitted bond length is in a good agreement of theoretical ones, while the coordination number (CN) shows a relatively large deviation from the theoretical ones. The CN (= 5.41) of Cr-N only shows a slight decrease compared with 6. However, the CN (= 8.80) of Cr-Cr is far from the theoretical number of 12. This result confirms from the local bonding aspect that the CrN film shows a Cr deficiency non-stoichiometric property.

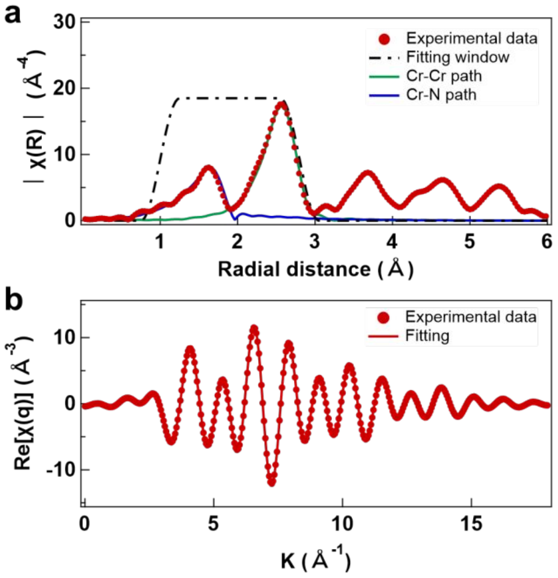

Fig. S13. (**a**) Fourier transformed EXAFS data and fitting results of the CrN thin film; (**b**) Experimental and simulated Back-Fourier transformed EXAFS spectra.

Table S1. Curve fitting results of Cr K-edge EXAFS in CrN thin films.

Path	Coordination Number, CN	Atomic distance (Å), R	Debye-Waller factor, σ^2	Reduced, χ^2
**Cr-Cr**	8.80± 0.636	2.95± 0.004	0.008	220
**Cr-N**	5.41± 0.657	2.08± 0.005	0.006	

14. Band structure of CrN and CrN'.

The band structure was studied to analyze the origin of semiconductor type differences. The absorption coefficient α was calculated from the following equation:[11]

$$\alpha = \ln\left[\frac{(1-R)^2+\{(1-R)^4+4R^2T^2\}^{\frac{1}{2}}}{2T}\right]/d \qquad \textbf{(S1)}$$

Where d is film thickness, T is transmittance, and R is reflectance. The bandgap E_g can be estimated from the α vs. wavelength curve using the Tauc plot method:[11]

$$(\alpha h\nu)^{1/n} = A(h\nu - E_g) \qquad \textbf{(S2)}$$

Where h is Planck constant, v is frequency, and A is a proportional constant. The value n, which equals 2 or 1/2, can determine the indirect and direct transition, respectively. The reflectance and transmittance spectra of CrN and CrN' films are shown in **Fig. S14a** and **b**. **Fig. S14c** shows the $(\alpha h\nu)^{1/2}$ as a function of $h\nu$ for CrN and CrN' thin films with a thickness around 100 nm, which exhibits an indirect transition in the Tauc plot. By extrapolating the linear region to abscissa yields, the bandgap was determined to be 0.18 eV for CrN' and 0.77 eV for CrN thin films, respectively. To understand the position of Fermi level in the band structure, the valence band spectra of the CrN and CrN' thin films were measured by HAXPES, as shown in **Fig. S14d** and **e**. The Fermi level relative to the valence band maximum (VBM) (E_F-E_v) was estimated to be 0.05 and 0.1 eV for CrN and CrN' thin films, respectively. The schematic of the band structures was depicted in the inset of **Fig. S14d** and **e**. In the CrN thin film, the fermi level locates near the valence band, indicating p-type conduction. While in CrN', the fermi level is closer to the conduction band, which is a typical n-type semiconductor. From the band structure, the intrinsic reason for different semiconducting types can be understood.

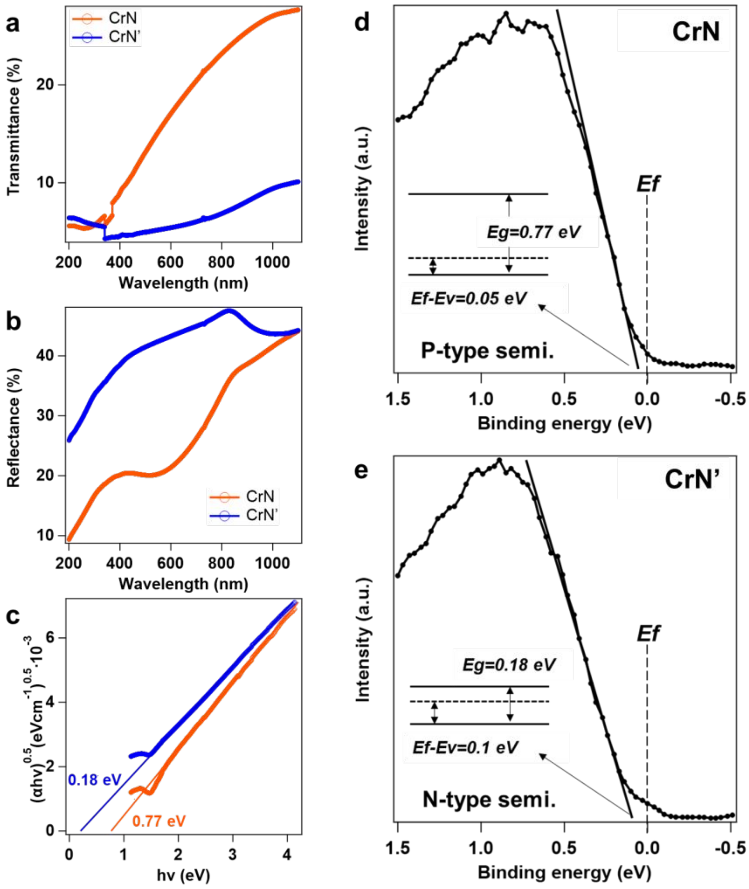

Fig. S14. The (**a**) transmittance, (**b**) reflectance and (**c**) absorption coefficient of CrN and CrN' thin film. The valance band maximum of (**d**) CrN and (**e**) CrN'

15. Thermal distribution simulation in CrN memory cell.

To understand the driving force of ion diffusion and phase change in CrN-based memory cells, the thermal and electrical physics of the memory devices were simply modeled without considering the thermoelectric effect (Thomson and Peltier effects), thermal boundary resistance, and electrical interface resistance. The CrN/metal electrode interfacial properties could also be ignored because the contact resistance has been confirmed to be a minor factor in the device operation and CrN behaves more like a metal than a semiconductor in conducting property terms. A constant voltage of 0.2 V was driven as a simulation input from the BE, and Joule heating was used to determine the heat generation throughout the structure. The simulations were based on the current conservation law and energy conservation law as follows:

$$\nabla \cdot (\sigma \nabla \emptyset) = 0, \quad \textbf{(S3)}$$

$$j = -\sigma \nabla \emptyset, \quad \textbf{(S4)}$$

and

$$\rho C_p \frac{\partial T}{\partial t} = \nabla \cdot (k \nabla T) + \rho_c (j \cdot j) \quad \textbf{(S5)}$$

where σ is the electrical conductivity, j is the current density, $\emptyset$ is the electrostatic potential, ρ is the density, C_p is the specific heat, *T* is the temperature, *t* is the time, *k* is the thermal conductivity, and ρ_c is the resistivity. The program that solves these equations is implemented in the open-source software, OpenFOAM. The corresponding values for CrN, TiN, W, and SiO_2 in the devices used for the calculation are listed in **Table S2**.

Table S2. Resistivity, thermal conductivity, density, and specific heat values used for the simulations.

	**Resistivity (mΩ·cm)**	**Thermal conductivity (W/mK)**	**Density (g/cm³)**	**Specific heat (J/mol·K)**
**CrN**	0.93	2.3[12]	5.9	52.44[13]
**TiN**	2.5E−2[14]	10[15]	5.4	37.22[13]
**W**	5.6E−3[16]	50[15]	19.25	24.31[13]
**SiO_2**	∞	1.38[15]	2.65	44.57[13]

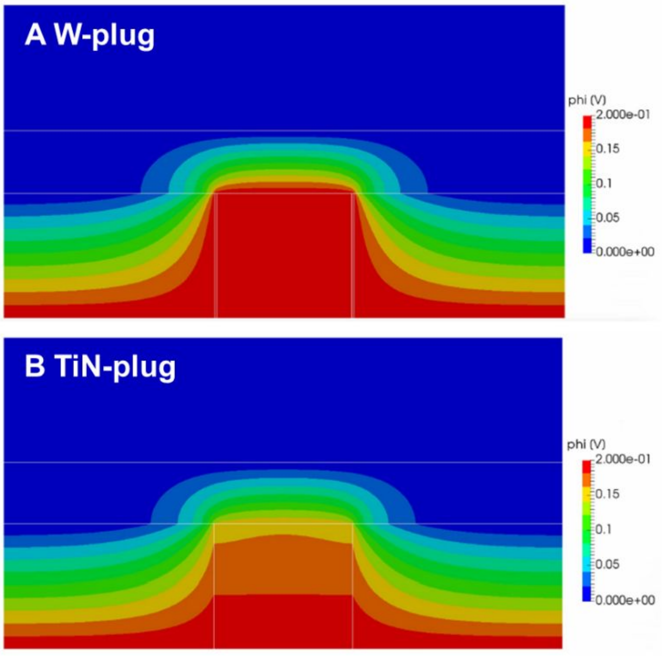

Fig. S15. Simulated electrical field distribution. In (**a**) W plug with a 4-nm TiN adhesion layer and (**b**) TiN plug devices.

16. Thermal stability of CrN_2 phase.

We measured the temperature dependence resistance change (R-T) of the memory device. Since the pad size of T-shape device is too small for the probes size in our probe-furnace system, we fabricated the new structure device with larger electrode pads (see the schematic device structure in the inset of **Fig. S16a**, and the fabrication flow in ref[17]). The memory cell was initially in a low resistance Set state and was Reset to a HRS (5×10^7 **Ω**) by applying voltage pulses as shown in **Fig. S16a**. Note that the smaller resistance contrast in this device is possibly due to the larger contact area (3×10^4 nm^2) between CrN and W electrode. The memory cell in high resistance Reset state was then transferred to the probe furnace. The furnace was firstly vacuumed to 10^{-1} Pa and the film was then annealed in a gradient temperature with a heating rate of 10 °C/min up to 400 °C under the Ar atmosphere. **Fig. S16b** shows R-T curve of the HRS CrN memory cell. The resistance decreased slightly with increasing temperature until the phase transition point ($T_{phase\ change}$) was reached, after which the resistance decreased sharply corresponding to a phase transition from CrN_2 to CrN. The $T_{phase\ change}$ was then determined to lie within the range of 250°C to 300°C by taking the minimum of the first derivative of the R-T curve, which is much larger than the crystallization temperature of traditional PCM: GST (~150 ºC).[18] Another CrN memory cell was also evaluated for the thermal stability as shown in **Fig. S16c** using the same method. The $T_{phase\ change}$ was found to be located in the range of 250°C to 300°C, emphasizing the thermal stability of the CrN_2 phase.

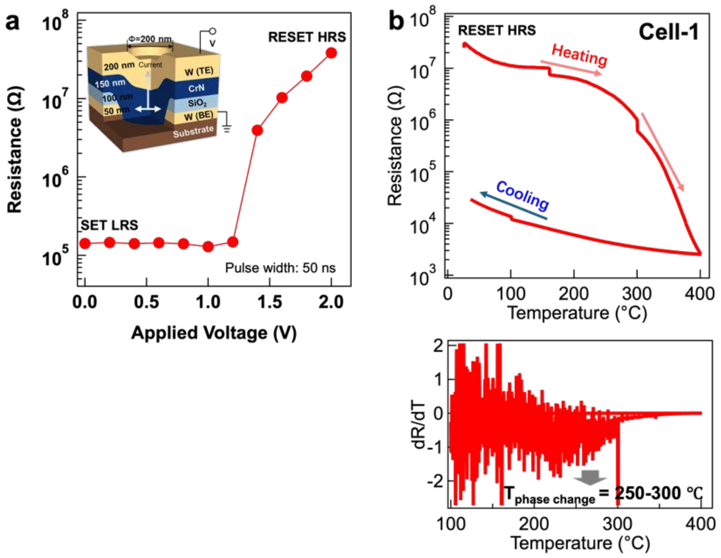

Fig. S16. (**a**) Resistance as a function of the pulse voltage during the Reset process of a CrN memory cell-1; (**b**) R-T curve of CrN memory cell-1 in the HRS; (**c**) R-T curve of the CrN memory cell-2 in the HRS.

17. Cyclic resistive switching of CrN memory.

We investigated the feasibility of cyclic resistive switching in both CrN-based memory devices (**Fig. S17a**). Although our devices exhibited resistive switching under unipolar positive voltage pulses, the narrow window of the pulse voltage applied for switching to the HRS (as small as 0.2 V) made it difficult to switch repeatedly under a constant pulse width. For example, the HRS was sometimes hard to achieve accurately because a little bit higher voltage could induce an LRS beyond the voltage limit for the HRS. In T-shaped PCRAM operation, positive electrical pulses are generally applied to the TE for both the RESET and SET processes because of the higher thermal efficiency compared with the case of negative electrical pulses applied to the BE [19]. Therefore, to investigate the endurance of the CrN-based memory devices, we adopted a negative electrical pulse direction to lower the thermal efficiency for the SET process, which enabled a large window of pulse voltage for the RESET/SET switch. The CrN-based memory device with a plug size of 45 nm × 45 nm showed bipolar switching. The device with an LSR of ~2×10^4 Ω started to switch to an HRS of ~2×10^8 Ω when applying a positive voltage pulse of 1.4 V, and it went back to the LRS after applying a negative voltage pulse of −1.4 V with a width of 50 ns (**Fig. S17b**).

Note that the programming window in the bipolar cyclic measurements was about 10^4, still limited compared with other PCMs such as GST. Thus, we investigated the failure mechanism of the CrN memory device for better cyclability. **Fig. S17c-e** shows the Reset/Set voltage dependence of the endurance properties. The endurance was tested using the same cell size (45 × 45 nm^2) with fixed pulse width 50 ns and read voltage 0.1 V. As shown in **Fig.S17c**, when the Set and Reset voltage were set to -2 V and 2 V, respectively, the memory cell could be cycled for more than 20 times with a HRS/LRS resistance contrast of around 10^5. When the Set and Reset voltages were reduced, the resistance contrast between HRS and LRS decreased to around 10^4, but with an improved cyclability of 10^3 (**Fig. S17d**). Further reducing the voltage of the Reset and Set process to -1 V and 1.2 V, respectively, over 10^4 cycles can be achieved, while the resistance contrast decreased to less than 10^3 (**Fig. S17e**). Our results indicate that the

resistance of both HRS and LRS strongly depends on the amplitude of the applied pulse voltage. Higher Reset voltage leads to a longer diffusion path of Cr and N atoms, resulting in a larger phase change volume and higher HRS resistance. Conversely, decreasing the applied Reset voltage decreases the phase change volume and HRS resistance simultaneously. When the Set voltage is further decreased, the high resistance CrN_2 volume cannot be fully Set back to CrN due to insufficient heat transformation, resulting in a higher resistance of LRS. Although larger Reset/Set voltage in our T-shape memory cell can enlarge the programing window, severe diffusion of Cr and N atoms under high voltage (Joule heating energy) makes it hard to self-heal after certain cycles of switching, thus accelerating the deterioration of the memory cell. Similar phenomena have been observed in traditional PCMs such as GST, which exhibits a strong Reset/Set energy dependence of endurance property due to unwanted atom migration in the T-shape device. The best cyclic property for both GST and GeTe is around $10^{6\sim9}$.[20,21] To improve endurance, various approaches have been proposed, such as engineering the bottom electrode[21] or utilizing a confined memory structure with a thin metallic liner to restrain atomic displacement[22]. However, the HRS/LRS resistance contrast would also be inevitably degraded with more cycles (e.g., On/off < 10; Cycle > 10^{12} for GST in a confined PCRAM).[6,23] Therefore, we believe that much better cyclic property can be obtained in the same confined PCRAM structure, and it will also be interesting to investigate the trade-off relationship of the on/off ratio and endurance in the new structure in our future work.

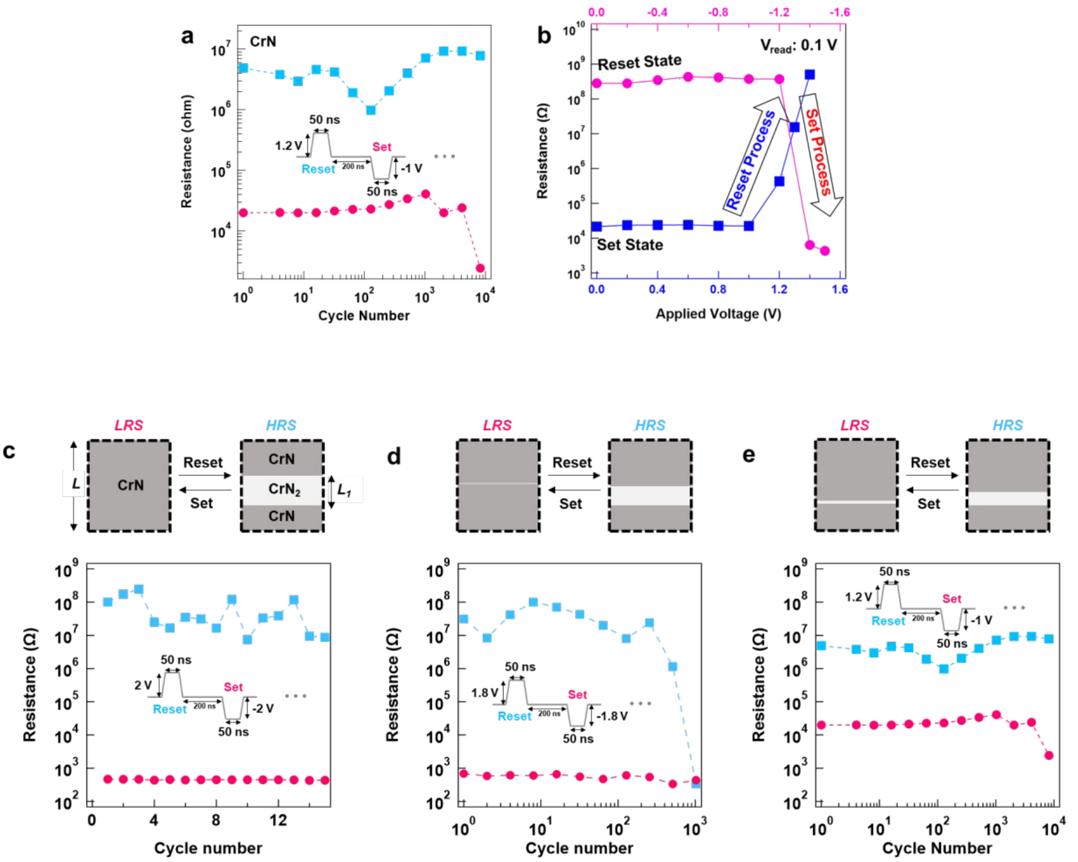

Fig. S17. (**a**) Results on endurance tests on cells with a plug size of 45 nm × 45 nm. (**b**) Bipolar pulse switching behavior of a CrN-based memory cell. *RV* characteristics for a plug size of 45 nm × 45 nm**.** (**c-e**) Endurance test of CrN device at various pulse conditions.

Table S3. The comparison of memory performance.

**PCM**	***Switching cycles***	***Switching speed***	***Scalability (Cell distance)***	***Programming window***	***Energy consumption***
CrN	10^4	30 ns	<40 nm	$10^{3\sim5}$	~100 pJ (T-Shape, 37×37nm)
GST[6,20,24]	$10^{5\sim12}$	50 ns	40 nm	$10^{1\sim2}$	~900 pJ (T-Shape, 37×37nm)
MnTe[25]	$10^{2\sim3}$	10 ns	<40 nm	$10^{2\sim3}$	<10%GST (Pore structure)

18. Transient current measurement.

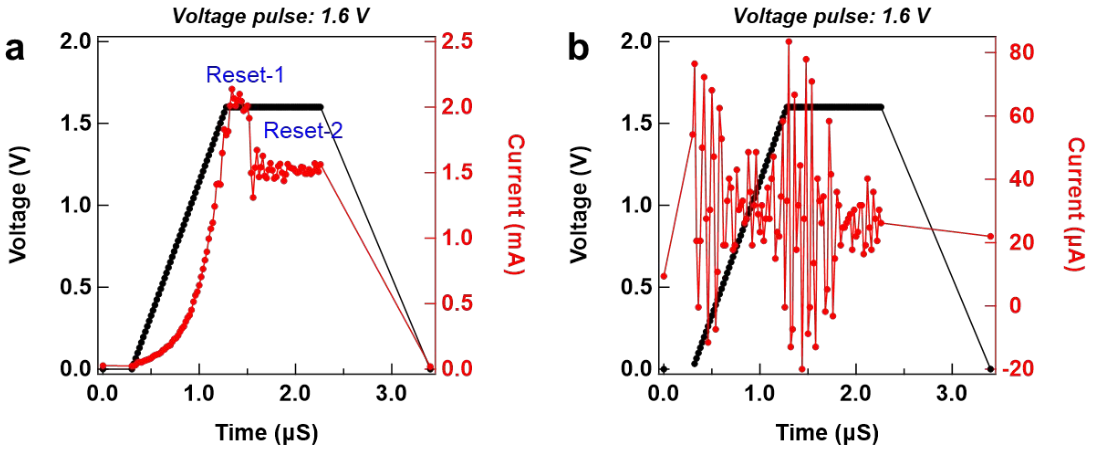

Fig. S18. Voltage and subsequent current flowing through the device (45 nm × 45 nm) when the Reset pulse is applied.

19. TEM observation in Set state of CrN memory device.

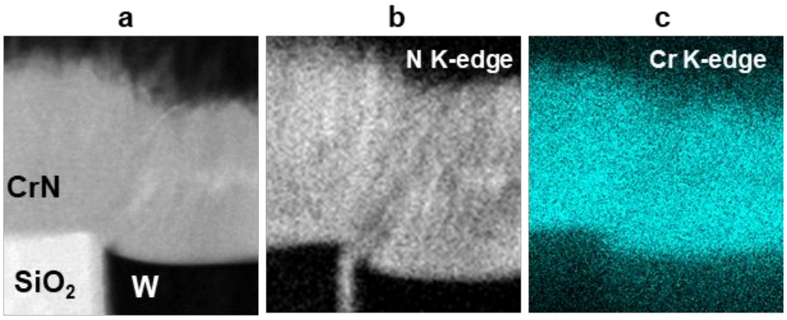

Fig. S19. (**a**) Cross-sectional TEM image of the CrN-based device (218 nm × 218 nm) in LRS Set state near the phase-change region. (**b**) Its corresponding electron energy loss spectroscopy mapping at the N *K*-edge. (**c**) Its corresponding energy-dispersive X-ray spectroscopy (EDX) mapping at Cr *K*-edge.

Reference

(1) Zambelli, C.; Navarro, G.; Sousa, V.; Prejbeanu, I. L.; Perniola, L. Phase Change and Magnetic Memories for Solid-State Drive Applications. *Proceedings of the IEEE* **2017**, *105* (9), 1790–1811.

(2) Wong, H. S. P.; Ahn, C.; Cao, J.; Chen, H. Y.; Fong, S. W.; Jiang, Z.; Neumann, C.; Qin, S.; Sohn, J.; Wu, Y. Stanford Memory Trends. *tech. report* **2016**.

(3) Min, K. P.; Li, C. Y.; Chang, T. J.; Chu, S. Y. The Effects of Si Doping on the Endurance and Stability Improvement of AlN-Based Resistive Random Access Memory. *ACS Appl Electron Mater* **2021**, *3* (12), 5327–5334.

(4) Simpson, R. E.; Fons, P.; Kolobov, a V; Fukaya, T.; Krbal, M.; Yagi, T.; Tominaga, J. Interfacial Phase-Change Memory. *Nat Nanotechnol* **2011**, *6* (July), 501–505.

(5) Ding, K.; Wang, J.; Zhou, Y.; Tian, H.; Lu, L. L.; Mazzarello, R.; Jia, C.; Zhang, W.; Rao, F.; Ma, E. Phase-Change Heterostructure Enables Ultralow Noise and Drift for Memory Operation. *Science (1979)* **2019**, *366* (6462), 210–215.

(6) Xie, Y.; Kim, W.; Kim, Y.; Kim, S.; Gonsalves, J.; BrightSky, M.; Lam, C.; Zhu, Y.; Cha, J. J. Self-Healing of a Confined Phase Change Memory Device with a Metallic Surfactant Layer. *Advanced Materials* **2018**, *30* (9), 1705587.

(7) Koch, C. DETERMINATION OF CORE STRUCTURE PERIODICITY AND POINT DEFECT DENSITY ALONG DISLOCATIONS. Doctor of Philosophy. ARIZONA STATE UNIVERSITY. USA. **2002**.

(8) Le Febvrier, A.; Gambino, D.; Giovannelli, F.; Bakhit, B.; Hurand, S.; Abadias, G.; Alling, B.; Eklund, P. P-Type Behavior of CrN Thin Films via Control of Point Defects. *Phys Rev B* **2022**, *105* (10), 104108.

(9) Ravel, B.; Newville, M. ATHENA, ARTEMIS, HEPHAESTUS: Data Analysis for X-Ray Absorption Spectroscopy Using IFEFFIT. In *Journal of Synchrotron Radiation*; International Union of Crystallography, 2005; Vol. 12, pp 537–541.

(10) Tripathi, Y.; Gupta, R.; Seema; Gupta, M.; Phase, D. M.; Rajput, P. Study of Phase Formulation in CrN Thin Films and Its Response to a Minuscule Oxygen Flow in Reactive Sputtering Process. *Thin Solid Films* **2019**, *670*, 113–121.

(11) Mori, S.; Sutou, Y.; Ando, D.; Koike, J. Optical and Electrical Properties of ¡-MnTe Thin Films Deposited Using RF Magnetron Sputtering. *Mater Trans* **2018**, *59* (9), 1506–1512.

(12) Quintela, C. X.; Podkaminer, J. P.; Luckyanova, M. N.; Paudel, T. R.; Thies, E. L.; Hillsberry, D. A.; Tenne, D. A.; Tsymbal, E. Y.; Chen, G.; Eom, C. B.; Rivadulla, F. Epitaxial CrN Thin Films with High Thermoelectric Figure of Merit. *Advanced Materials* **2015**, *27* (19), 3032–3037.

(13) Webbook, N.; Nist, T.; Webbook, C.; Reference, S.; Program, D. Welcome to the NIST Chemistry WebBook. *Secretary* **2012**, 1–2.

(14) Sosnin, D.; Kudryashov, D.; Mozharov, A. Investigation of Electrical and Optical Properties of Low Temperature Titanium Nitride Grown by Rf-Magnetron Sputtering. *J Phys Conf Ser* **2017**, *917* (5), 052025.

(15) Ahn, C.; Fong, S. W.; Kim, Y.; Lee, S.; Sood, A.; Neumann, C. M.; Asheghi, M.; Goodson, K. E.; Pop, E.; Wong, H. S. P. Energy-Efficient Phase-Change Memory with Graphene as a Thermal Barrier. *Nano Lett* **2015**, *15* (10), 6809–6814.

(16) Elert, G. *Resistivity of Gold, The Physics Factbook.* https://hypertextbook.com/facts/2004/DeannaStewart.shtml (accessed 2021-07-08).

(17) Shuang, Y.; Sutou, Y.; Hatayama, S.; Shindo, S.; Song, Y. H.; Ando, D.; Koike, J. Contact Resistance Change Memory Using N-Doped $Cr_2Ge_2Te_6$ Phase-Change Material Showing Non-Bulk Resistance Change. *Appl Phys Lett* **2018**, *112* (18),

(18) Yamada, N.; Ohno, E.; Nishiuchi, K.; Akahira, N.; Takao, M. Rapid-Phase Transitions of GeTe-Sb_2Te_3 Pseudobinary Amorphous Thin Films for an Optical Disk Memory. *J Appl Phys* **1991**, *69* (5), 2849–2856.

(19) Hatayama, S.; Song, Y. H.; Sutou, Y. Low Resistance-Drift Characteristics in $Cr_2Ge_2Te_6$-Based Phase Change Memory Devices with a High-Resistance Crystalline Phase. *Mater Sci Semicond Process* **2021**, *133*, 105961.

(20) Ding, K.; Wang, J.; Zhou, Y.; Tian, H.; Lu, L. L.; Mazzarello, R.; Jia, C.; Zhang, W.; Rao, F.; Ma, E. Phase-Change Heterostructure Enables Ultralow Noise and Drift for Memory Operation. *Science (1979)* **2019**, *366* (6462), 210–215.

(21) Wu, J. Y.; Breitwisch, M.; Kim, S.; Hsu, T. H.; Cheek, R.; Du, P. Y.; Li, J.; Lai, E. K.; Zhu, Y.; Wang, T. Y.; Cheng, H. Y.; Schrott, A.; Joseph, E. A.; Dasaka, R.; Raoux, S.; Lee, M. H.; Lung, H. L.; Lam, C. A Low Power Phase Change Memory Using Thermally Confined TaN/TiN Bottom Electrode. *Technical Digest - International Electron Devices Meeting, IEDM* **2011**.

(22) Lama, G.; Bourgeois, G.; Bernard, M.; Castellani, N.; Sandrini, J.; Nolot, E.; Garrione, J.; Cyrille, M. C.; Navarro, G.; Nowak, E. Reliability Analysis in GeTe and GeSbTe Based Phase-Change Memory 4 Kb Arrays Targeting Storage Class Memory Applications. *Microelectronics Reliability* **2020**, *114*.

(23) Kim, W.; Brightsky, M.; Masuda, T.; Sosa, N.; Kim, S.; Bruce, R.; Carta, F.; Fraczak, G.; Cheng, H. Y.; Ray, A.; Zhu, Y.; Lung, H. L.; Suu, K.; Lam, C. ALD-Based Confined PCM with a Metallic Liner toward Unlimited Endurance. *Technical Digest - International Electron Devices Meeting, IEDM* **2017**, 4.2.1-4.2.4.

(24) Ha, T. J.; Shin, S.; Keun Kim, H.; Hong, M. H.; Park, C. S.; Hee Cho, H.; Jin Choi, D.; Park, H. H. Use of Ordered Mesoporous SiO_2 as Protection against Thermal Disturbance in Phase-Change Memory. *Appl Phys Lett* **2013**, *102* (14), 144102.

(25) Mori, S.; Hatayama, S.; Shuang, Y.; Ando, D.; Sutou, Y. Reversible Displacive Transformation in MnTe Polymorphic Semiconductor. *Nat Commun* **2020**, *11* (1).
